# New Insights into the Anti-pathogenic Potential of *Lactococcus garvieae* against *Staphylococcus aureus* Based on RNA Sequencing Profiling

**DOI:** 10.3389/fmicb.2017.00359

**Published:** 2017-03-08

**Authors:** Pierre Delpech, Etienne Rifa, Graham Ball, Sabine Nidelet, Emeric Dubois, Geneviève Gagne, Marie-Christine Montel, Céline Delbès, Stéphanie Bornes

**Affiliations:** ^1^Université Clermont Auvergne, INRA, UMRFAurillac, France; ^2^John van Geest Cancer Research Centre, School of Science and Technology, Nottingham Trent UniversityNottingham, UK; ^3^Montpellier GenomiX, Institut de Génomique FonctionnelleMontpellier, France

**Keywords:** *Lactococcus garvieae*, *Staphylococcus aureus*, hydrogen peroxide, transcriptome, growth inhibition, anti-pathogenic interactions

## Abstract

The bio-preservation potential of *Lactococcus garvieae* lies in its capacity to inhibit the growth of *staphylococci*, especially *Staphylococcus aureus*, in dairy products and *in vitro*. *In vitro*, inhibition is modulated by the level of aeration, owing to hydrogen peroxide (H_2_O_2_) production by *L. garvieae* under aeration. The *S. aureus* response to this inhibition has already been studied. However, the molecular mechanisms of *L. garvieae* underlying the antagonism against *S. aureus* have never been explored. This study provides evidence of the presence of another extracellular inhibition effector *in vitro*. This effector was neither a protein, nor a lipid, nor a polysaccharide, nor related to an L-threonine deficiency. To better understand the H_2_O_2_-related inhibition mechanism at the transcriptome level and to identify other mechanisms potentially involved, we used RNA sequencing to determine the transcriptome response of *L. garvieae* to different aeration levels and to the presence or absence of *S. aureus*. The *L. garvieae* transcriptome differed radically between different aeration levels mainly in biological processes related to fundamental functions and nutritional adaptation. The transcriptomic response of *L. garvieae* to aeration level differed according to the presence or absence of *S. aureus*. The higher concentration of H_2_O_2_ with high aeration was not associated with a higher expression of *L. garvieae* H_2_O_2_-synthesis genes (*pox*, *sodA*, and *spxA1*) but rather with a repression of *L. garvieae* H_2_O_2_-degradation genes (*trxB1*, *ahpC*, *ahpF*, and *gpx*). We showed that *L. garvieae* displayed an original, previously undiscovered, H_2_O_2_ production regulation mechanism among bacteria. In addition to the key factor H_2_O_2_, the involvement of another extracellular effector in the antagonism against *S. aureus* was shown. Future studies should explore the relation between H_2_O_2_-metabolism, H_2_O_2_-producing LAB and the pathogen they inhibit. The nature of the other extracellular effector should also be determined.

## Introduction

Interest in Lactic Acid Bacteria (LAB) as bio-preservation agents against foodborne pathogens has been growing for the last 15 years (Ito et al., 2003; Batdorj et al., 2007; Charlier et al., 2009; Adesokan et al., 2010). Considering their beneficial properties, the dairy industry could find it useful to employ *Lactococcus garvieae* strains as starter or adjunct cultures, provided the strains are safe (Fernández et al., 2010). Aquatic strains of *L. garvieae* are frequently referenced as fish pathogens (Eldar and Ghittino, 1999; Vendrell et al., 2006) but other strains are regarded as opportunistic pathogens for humans (Aguado-Urda et al., 2011; Ortiz et al., 2014). Despite its ubiquity in foods such as milk (Devriese et al., 1999; Villani et al., 2001; Callon et al., 2007) and dairy products of various origins (Flórez and Mayo, 2006; Fortina et al., 2007; Alomar et al., 2008b; El-Baradei et al., 2008; Jokovic et al., 2008; Alegría et al., 2009; Sip et al., 2009; Monfredini et al., 2012; Pangallo et al., 2014; Morandi et al., 2015), to our knowledge, *L. garvieae* has never been involved in a foodborne disease outbreak. *L. garvieae* is of interest for bio-preservation owing to its ability to inhibit the growth of staphylococci, particularly *Staphylococcus aureus*, as has been observed in milk, in cheese and *in vitro* (Alomar et al., 2008a,b; Delbes-Paus et al., 2010; Delpech et al., 2015). The transcriptome response of *S. aureus* to this inhibition has already been explored (Delpech et al., 2015). It is associated with a repression of the stress response (especially H_2_O_2_ response) and of cell division genes and with modulation of the expression of virulence genes (particularly *agrA*, *hld*, and enterotoxin-encoding genes) genes. However, the molecular mechanisms of *L. garvieae* underlying the antagonism against *S. aureus* have never been explored.

Since *L. garvieae* produces low amounts of acetic and lactic acid in milk (Alomar et al., 2008a,b; Nouaille et al., 2009; Sip et al., 2009; Delbes-Paus et al., 2010; Delpech et al., 2015; Morandi et al., 2015), its antagonism against *S. aureus* is not associated with acidification. It is also unlikely to be associated with nutritional competition (Alomar et al., 2008a). With high aeration, *S. aureus* inhibition is mainly associated with hydrogen peroxide (H_2_O_2_) production by *L. garvieae*, as already observed *in vitro* (Delbes-Paus et al., 2010; Delpech et al., 2015). With low aeration, the inhibition is weaker and H_2_O_2_ is not detected. The addition of catalase (an H_2_O_2_-degrading enzyme) partly suppressed the inhibition (Delbes-Paus et al., 2010). Delbes-Paus et al. (2010) therefore suggested that at least one other molecule from *L*. *garvieae* must be involved in the residual inhibition of *S. aureus* in the absence of H_2_O_2_. The role and nature of this molecule have not yet been determined.

In Gram-positive bacteria, the aeration level and the presence of H_2_O_2_ generally strongly affect the expression of H_2_O_2_ metabolism genes. The expression of major H_2_O_2_ degradation genes (*ahpC*, *ahpF*, *gshR*, *gpo*, *trxA*, and *trxB*) and their related proteins is strongly induced in *L. lactis* (Pedersen et al., 2008) under aeration and in *Bacillus subtilis* in the presence of H_2_O_2_ (Mostertz et al., 2004). As regards the major H_2_O_2_ synthesis genes, *sodA* is generally regulated in the same way as H_2_O_2_-degradation genes while the pyruvate oxidase (*pox*) gene is not affected by these parameters. Biological functions of *L. lactis* related to aerobiosis, i.e., O_2_ response, menaquinone metabolism and stress response, are variably affected by the presence of *S. aureus* (Nouaille et al., 2009). However, little is known about the H_2_O_2_-metabolism of *L. garvieae* as an anti-pathogenic process.

In this study, we aimed to characterize the antagonism of *L. garvieae* against *S. aureus in vitro* in greater depth. Firstly, we investigated the presence of potential antagonist molecules in H_2_O_2_-free *L. garvieae* supernatants and their impact on *S. aureus* growth. Secondly, the transcriptome of *L. garvieae* with different aeration levels and in different biotic environments (absence or presence of *S. aureus*) was determined through RNA sequencing, an accurate and efficient method for revealing bacterial transcriptome profiles (Pinto et al., 2011). The resulting data led us to a better understanding of *L. garvieae* hydrogen peroxide metabolism and to some initial hypotheses on the nature of a new inhibition effector.

## Materials and Methods

### Strains and Culture Conditions

*Lactococcus garvieae* N201 and *S. aureus* SA15, isolated from raw milk, were obtained from the INRA UR545 collection (Alomar et al., 2008a). Both strains were aerobically grown in Brain-Heart Infusion broth (“BHI”, Biokar Diagnostic, Pantin, France) for 20 h, at 30°C for *L. garvieae* and at 37°C for *S. aureus*. They were then inoculated separately or in co-culture at 10^6^ cells.mL^-1^ for *S. aureus* and 10^7^ cells.mL^-1^ for *L. garvieae* into BHI buffered at pH = 7 with phosphate buffer KH_2_PO_4_, 3H_2_O/K_2_HPO_4_ at 0.1 mol.L^-1^ (KH_2_PO_4_, 3H_2_O, Riedel-de-Haen, Honeywell GmbH, Seelze, Germany; K_2_HPO_4_, Merck KGaA, Darmstadt, Germany) previously equilibrated at 30°C. Pure cultures and co-cultures of both strains were performed with either a high or a low aeration depending on the experiment. Low aeration cultures were set in static, fully filled and sealed, 50-mL Nunc EZ Flip conical centrifuge tubes (Sigma–Aldrich, St. Louis, MO, USA). High aeration was obtained by a mechanical shaking at 150 rpm on 50-mL cultures in 250-mL Erlenmeyers. All cultures were incubated at 30°C for 24 h in an Infors HT Minitron (Infors AG, Bottmingen, Switzerland). The cultivable cell counts were determined after plating for each sampling time as described by Delpech et al. (2015).

### Impact of H_2_O_2_-Free Co-culture Supernatants on *S. aureus* Growth

The potential presence of other antistaphylococcal molecules in *L. garvieae* N201 and *S. aureus* SA15 co-culture supernatant and their impact on *S. aureus* planktonic growth were investigated. After adding catalase at 400 U.mL^-1^, a pure culture of *S. aureus* SA15, a co-culture of *S. aureus* SA15 and *L. garvieae* N201 and plain BHI were incubated with low aeration as described above. After 6 h of incubation, 40 mL of each tube were centrifuged at 9,600 × *g* for 10 min at 4°C and supernatants stored at 4°C until utilization. Four milliliters of the remaining *S. aureus* culture were centrifuged at 7,500 × *g* for 10 min. The cell pellet was resuspended at a concentration of ∼10^6^ cells.mL^-1^ in 20 mL of fresh 2X-concentrated BHI buffered at pH = 7. The supernatants were filtered through a cellulose acetate membrane (pore size 0.45 μm; GVS S.p.A., Zola Predosa, Italy). They were either treated with proteinase K (AMRESCO LLC, Solon, OH, USA, ref: 0706-100MG) and pronase E (Merck KGaA, ref: 537088), i.e., with two proteases, or treated with lipase (Sigma–Aldrich, ref: L3126-100G), or treated with α-amylase (Sigma–Aldrich, ref: A3176-500KU), or not treated at all. Each enzyme was added at 0.2 mg.mL^-1^ from stock solutions at 10 mg.mL^-1^ prepared in 200 mM of phosphate buffer at pH = 7. Treatment with proteases consisted of a first incubation of the supernatants with proteinase K at 50°C for 2 h 15 and a second incubation with pronase E at 37°C for 2 h 15. Treatments with lipase and α-amylase consisted of an incubation of the supernatants with the enzyme at 37°C for 5 h. After each incubation step, the enzyme was inactivated by heating (95°C during 10 min) and all supernatants were filtered again through a cellulose acetate membrane (pore size 0.45 μm; GVS S.p.A.). Prepared supernatants and *S. aureus* culture in 2X-BHI were then distributed at 1:1 volume ratio (total volume = 1.5 mL) in a CytoOne 24-well cell plate covered with a lid (ref: CC7672-7524; STARLAB, Hambourg, Germany). The cell plate was incubated for 25 h in a SAFAS Xenius XC spectrophotometer (SAFAS Monaco, Monaco) and thermostated at 30°C with a Julabo CryoStat (JULABO Gmbh, Seelbach, Germany). *S. aureus* growth in each well was determined by measuring the OD_600_ every 15 min for 1 h and then every 30 min for 24 h. Before each OD_600_ measurement, the cell plate was shaken at 5 Hz for 20 s with an orbital diameter of 6 mm. The whole experimental design was repeated three times. To identify sample means that were significantly different from each other, statistical analyses were performed on values at 9, 12, 15, 18, 21, and 24 h, using Statistica software (StatSoft) with a single-factor analysis of variance (ANOVA) followed by a Newman–Keuls *post hoc* test.

### Sample Preparation for RNA Analysis

Pure cultures and co-cultures of *L. garvieae* N201 and *S. aureus* SA15 were grown with high or low aeration, as specified above. After 3, 6, 9, and 24 h of incubation, 40 mL of each culture were centrifuged at 9,600 × *g* for 10 min at 4°C. Hydrogen peroxide concentrations and pH values were determined on the supernatants by enzymatic reaction and spectrophotometry as described by Delbes-Paus et al. (2010). The cell pellets were immediately frozen in an ethanol bath and stored at -80°C. Extraction of total RNA from the frozen cell pellets was performed as described by Delpech et al. (2015). For each sample of total RNA obtained with one culture condition, a first part of the aliquot was used for RNA sequencing after rRNA depletion and a second part of the aliquot was used for RT-qPCR analyses. The whole experimental design was repeated three times.

### Determination of *L. garvieae* Transcriptome Changes by RNA Sequencing

Ribosomal RNAs were removed from the total RNA (2 × 5 μg of RNA by sample) using a RiboZero Magnetic Kit for Gram Positive Bacteria (Illumina Inc., San Diego, CA, USA) according to the manufacturer’s instructions. The quality and concentration of RNA in each sample were assessed using a RNA 6000 pico kit (Agilent Technologies, Santa Clara, CA, USA). The following steps were performed by the MGX Platform (Montpellier GenomiX, CNRS, Montpellier, France) using Illumina kits and devices (Illumina Inc.): construction of the mRNA library using a TruSeq Stranded mRNA Sample Preparation Kit; cluster generation with the cBot system using a Cluster Generation Kit; hybridization of the sequencing primer on the flow-cell; 50-bp single-read sequencing using a HiSeq 2000 device with SBS technology; informatic pretreatments, i.e., image analysis with the HiSeq Control Software and Real-Time Analysis component, base-calling with the RTA software and demultiplexing with CASAVA (Illumina). The RNAseq data are available from NCBI GEO datasets under the accession number GSE74030.

The quality scores across all bases of all reads and the N (non-attributed bases) content across all bases were determined for each condition with the FastQC software from the Babraham Institute^1^. Both analyses showed good quality values (data not shown). The reads were next aligned simultaneously on reference genomes, i.e., *L. garvieae* N201 (unpublished, GOLD project ID = Gp0034836 and NCBI BioProject ID = 184287) and *S. aureus* MW2 (RefSeq number = NC_003923.1), using the BWA package (Li and Durbin, 2009) with a seed of 32 bases and a maximum of two mismatches tolerated on the seed. Using the Samtools suite (Li et al., 2009) we excluded from further analyses those reads with low alignment quality scores (MAPQ index < 20). Reads which mapped on multiple sites (between 0.3 and 3.2% depending on the sample) and reads which did not map on any site considering the stringency applied (between 8.7 and 15.5% depending on the sample) were excluded from further analysis (see Supplementary Table 1). Reads overlapping genes were counted with the HTSeq Count software in Union mode (Anders et al., 2015). Differentially expressed genes were identified using the Bioconductor R^2^ packages EdgeR, DESeq and DESeq2 (Anders and Huber, 2010; Robinson et al., 2010; Love et al., 2014). Genes with less than 15 reads (from the three biological replicates of two compared samples) were filtered and thus removed from the analysis. Data were normalized using the Relative Log Expression (RLE) normalization factor for EdgeR and the DESeq normalization factor for DESeq and DESeq2. Gene expression changes with adjusted *p*-value of less than 0.05 (by the FDR method from Benjamini–Hochberg) were declared differentially expressed. Differentially expressed genes highlighted by at least one of the three packages were considered for further investigation.

Genes were sorted into two lists: one of genes differentially expressed in both pure culture and co-culture and one of genes differentially expressed only in pure culture or only in co-culture. These lists were separately subjected to a Blast2Go analysis (Conesa et al., 2005). Blast2Go analysis consisted first of a BlastX^3^ on the nr database with 20 hits and a maximum *E*-value of 1.0*E*^-15^. The resulting data were enriched with an InterPro scan analysis (Zdobnov and Apweiler, 2001). Blast hits of each sequence were then mapped with Gene Ontology terms (Gene Ontology Consortium, 2008) annotated with a maximum *E*-value of 1.0*E*^-6^, a cut-off of 55 and a GO weight of 5. The number of genes involved in each Biological Process GO category was calculated from combined graphs with no filter and a score alpha of 0.6. Since more than 450 different biological processes were identified for each category of genes, we excluded biological processes involving less than nine genes. When several biological processes involved the same genes and were associated with comparable functions, we considered only the one most relevant to our scientific hypotheses.

For a deeper analysis of *S. aureus* direct effect on *L. garvieae* gene expression regardless of aeration level, data mining was undertaken using the regression functions in Microsoft excel. Expression values were systematically plotted for each pair of samples (co-culture versus pure culture), a standard residual value was determined from the regression analysis for each gene across multiple pair wise comparisons and the mean value was determined. This mean standard residual value was used to rank the genes and identify the strongest gene expression differences. A standard residual cut-off of 5 was used as the threshold for significance to account for false discovery using a Bonferroni correction and approximating the standard residual to an equivalent *p*-value for the number of comparisons.

### Determination of Gene Expression by RT-qPCR

Total RNA was retro-transcribed using a High Capacity cDNA Reverse Transcription kit (Invitrogen, Life Technologies, Carlsbad, CA, USA) following the supplier’s instructions. Ct of genes of interest (see Supplementary Table 2) were determined by RT-qPCR as described by Delpech et al. (2015). All primers were designed using PrimerExpress^®^ software (Applied Biosystems^®^, Life Technologies). By comparison with the Ct of the *tufB* reference gene stable in our conditions (data not shown), expression of a gene of interest (“goi”) was calculated using the formula introduced by Pfaffl (2001).

The influence of two experimental factors was studied: the presence of *S. aureus* (with high or low aeration, gene expression in co-culture divided by gene expression in pure culture) and aeration level (in presence or absence of *S. aureus*, gene expression in shaken condition divided by gene expression in static condition). Statistical analyses were performed using Statistica software (StatSoft, Inc., StatSoft France, Maisons-Alfort, France) by single-factor ANOVA followed by a Newman–Keuls *post hoc* test.

## Results

### Effect of Enzyme-Treated Co-culture Supernatants on *S. aureus* Planktonic Growth

Since the inhibition of *S. aureus* may not be related solely to hydrogen peroxide, we looked for another possible anti-staphylococcal molecule produced by *L. garvieae* in the culture supernatants. We monitored *S. aureus* growth (OD_600_) over 24 h in either non-inoculated BHI or in supernatants prepared from H_2_O_2_-free cultures (presence of catalase) with low aeration (either pure culture of *S. aureus* or co-culture of *L. garvieae* and *S. aureus*). In order to avoid any nutritional competition, BHI at a final concentration of 1X was added to each culture.

Growth of *S. aureus* in supernatant from *S. aureus* pure culture was comparable to that in plain BHI (data not shown). During the stationary phase, the OD_600_ measured in *S. aureus* cultures in supernatant from co-culture was lower than that in supernatant from *S. aureus* pure culture (see Data Sheet 1). This inhibition was still observed when these supernatants were treated with proteases, lipase or α-amylase, the main macromolecule-degrading enzymes. pH values remained between 6.9 and 7.1 in all cultures (data not shown).

### Determination of Aeration and *S. aureus* Effects on *L. garvieae* Transcriptome by RNA Sequencing

To identify the best conditions for studying the transcriptome of *L. garvieae* N201 with respect to its capacity to inhibit *S. aureus* SA15, we followed the growth of the two bacteria in pure cultures and co-cultures in BHI for 24 h under high or low aeration conditions (**Table 1**).

**Table 1:** Cell counts and H_2_O_2_ concentration over 24 h in cultures of *Lactococcus garvieae* N201 and *Staphylococcus aureus* SA15 with high or low aeration levels.

Aeration level	Culture	*L. garvieae* cell concentration (log [CFU.mL^-1^])					*S. aureus* cell concentration (log [CFU.mL^-1^])					Hydrogen peroxide concentration (mM)				
		0 h	3 h	6 h	9 h	24 h	0 h	3 h	6 h	9 h	24 h	0 h	3 h	6 h	9 h	24 h
High	N201	6.6^a^	7.3^a^	8.4^a^	9.0^a^	8.6^a^	NT	NT	NT	NT	NT	ND	NT	0.5^a^	1.7^a^	1.5^a^
	SA15	NT	NT	NT	NT	NT	5.6^a^	5.2^ab^	6.1^a^	7.4^a^	9.0^a^	ND	NT	ND	ND	ND
	N201 + SA15	6.5^a^	7.4^a^	8.5^a^	8.6^a^	8.7^a^	5.6^a^	4.8^a^	3.9^b^	3.4^b^	4.5^b^	ND	NT	0.5^a^	1.6^a^	1.5^a^
Low	N201	6.6^a^	7.4^a^	8.4^a^	8.7^a^	8.9^a^	NT	NT	NT	NT	NT	ND	NT	ND	ND	ND
	SA15	NT	NT	NT	NT	NT	5.6^a^	6.1^c^	7.0^a^	7.3^a^	8.0^c^	ND	NT	ND	ND	ND
	N201 + SA15	6.5^a^	7.3^a^	8.8^a^	8.8^a^	8.9^a^	5.6^a^	5.6^bc^	6.2^a^	6.5^a^	6.2^d^	ND	NT	ND	ND	ND

*In the same column, letters (a, b, and c) indicate homogeneous statistical groupings (*p*-value < 0.05 by Newman–Keuls method). ND, non-detectable values (below the spectrometry detection limit). NT, not tested*.

The growth of *L. garvieae* was not affected by *S. aureus*. The growth of *S. aureus* was inhibited by *L. garvieae* at both aeration levels. With high aeration, maximal inhibition was observed from 9 to 24 h (difference with pure culture of 4 and 4.5 log CFU/ml, respectively, at 9 and 24 h). At 9 h, H_2_O_2_ concentration reached a peak concomitant with the lowest *S. aureus* concentration. With low aeration, inhibition was weaker than with high aeration and was observed later, at 24 h. Concomitantly, H_2_O_2_ was not detected in these cultures. pH values remained between 6.9 and 7.1 in all cultures (data not shown).

Considering these data, we analyzed the *L. garvieae* transcriptome in pure culture and in co-culture with *S. aureus* after 9 h of incubation with high and the low aerations. RNA sequencing generated a number of reads per sample, ranging from 12,365,133 to 15,670,131 depending on the sample after the initial quality filter (see Supplementary Table 1). Between 83.1 and 91.0% of these reads mapped correctly onto the reference genomes. Considering the direct effect of *S. aureus* on *L. garvieae* gene expression, analysis of the mapped reads using the Bioconductor R packages failed to identify expression differences (data not shown). Additional regression analyses highlighted 39 genes differentially expressed in presence of *S. aureus*, of which 4 genes under low aeration and 35 genes under high aeration conditions (see Supplementary Table 3). The expression of ∼18% of *L. garvieae* genes, i.e., 358 genes, differed between the two aeration levels (see Supplementary Table 4). Among these 358 genes, the expression of 181 genes differed regardless of the presence or absence of *S. aureus* (i.e., similarly in pure culture and in co-culture). The expression of 177 *L. garvieae* genes responded differently to different aeration levels, depending on the presence or absence of *S. aureus*: 88 gene expressions differed only in pure culture and 89 gene expressions differed only in co-culture.

### Effect of Aeration Level on *L. garvieae* Biological Processes

**FIGURE 1:**
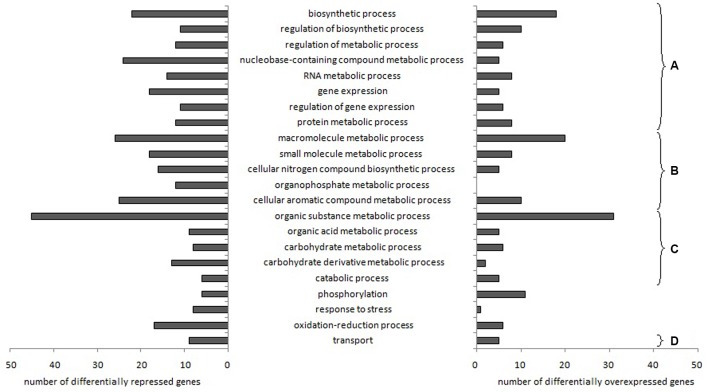
***Lactococcus garvieae* biological processes involving genes differentially expressed depending on aeration level in both pure culture and co-culture**. Bars represent the number of genes significantly overexpressed (left) or repressed (right) with high aeration compared to low aeration, according to at least one of the three R packages used (EdgeR, DESeq, and DESeq2). After the Blast2Go analysis and data filtering, biological processes were manually sorted into several categories: **(A)** processes related to fundamental growth function, i.e., global cellular, metabolic and biosynthetic processes, (post-)transcriptional and (post-)translational functions, **(B)** processes potentially associated with fundamental growth functions but also potentially associated with other metabolisms, **(C)** nutrition-related processes and **(D)** transport processes.

After RNA sequencing data treatments, 22 biological processes (named according to Gene Ontology termes) related to *L. garvieae* genes differentially expressed depending on aeration level in both pure culture and co-culture were identified using Blast2Go (**Figure 1**). Changes in gene expression are shown in Supplementary Table 4.

Most of the biological processes affected were related to fundamental growth functions (**Figures 1A,B**) and nutrition (**Figure 1C**). With high aeration, two genes (*ilvA*, LCGN_1922) related to “threonine metabolism” were repressed while two genes (LCGN_1919, LCGN_1920) were over-expressed.

Fourteen genes related to “transport” processes were differently expressed depending on aeration level both in pure culture and co-culture, including genes involved in the transport of metals (lead, cadmium, zinc, copper and/or mercury) and vitamins (riboflavin and folate).

Genes and GO biological processes related to O_2_ and H_2_O_2_ metabolism were also affected. “Oxidation-reduction process” and “response to stress” biological processes involved more repressed genes (17 and 8, respectively) than over-expressed genes (6 and 1, respectively). Three genes related to H_2_O_2_ metabolism (*ahpF*, *pox*, and *spxA1*) and one gene related to O_2_ consumption (*lox*) were repressed with the high aeration level. Several stress response genes (*hrcA*, *groES*, *groEL*, *dnaK*, *dnaJ*, *grpE*, *clpB*, and five genes belonging to the universal stress protein family) were repressed. Genes related to peroxide resistance (*ohrA* and *ohrR*) were strongly over-expressed under high aeration conditions. Electron Transport Chain (ETC) genes (*cydB*, *menH*, and *ubiE*) were over-expressed.

### Modulation by *S. aureus* of *L. garvieae* Response to Aeration

**FIGURE 2:**
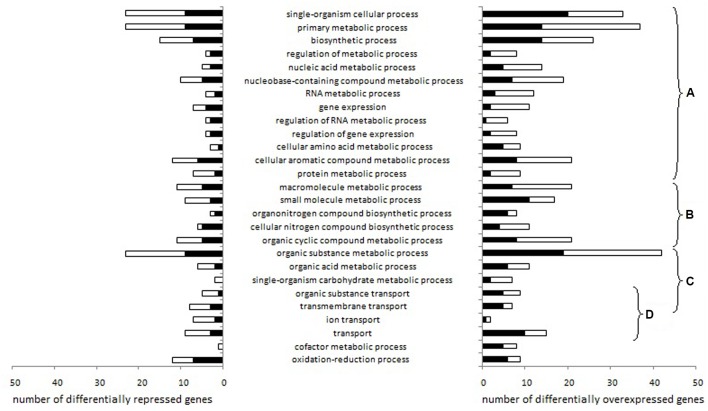
***Lactococcus garvieae* biological processes involving genes differentially expressed depending on aeration level exclusively in pure culture or exclusively in co-culture**. Stacked bars represent the number of genes significantly overexpressed (left) or repressed (right) with high aeration compared to low aeration in pure culture only (black bars) or in co-culture only (white bars), according to at least one of the three R packages used (EdgeR, DESeq, and DESeq2). After the Blast2Go analysis and data filtering, biological processes were manually sorted into several categories: **(A)** processes related to fundamental growth function, i.e., global cellular, metabolic and biosynthetic processes, (post-)transcriptional and (post-)translational functions, **(B)** processes potentially associated with fundamental growth functions but also potentially associated with other metabolisms, **(C)** nutrition-related processes and **(D)** transport processes.

We identified 27 Gene Ontology biological processes related to genes differentially expressed depending on aeration level either in pure culture or in co-culture (**Figure 2**).

Changes in gene expressions are shown in Supplementary Table 4. Most of the biological processes affected were related to fundamental growth functions (**Figures 2A,B**) or nutrition (**Figure 2C**). Under high aeration conditions, four lysine metabolism genes (LCGN_0575, LCGN_0576, LCGN_0577, LCGN_0578) and two threonine metabolism genes (LCGN_0576 and LCGN_0577) were over-expressed exclusively in pure culture while four galactose metabolism genes were over-expressed exclusively in co-culture (LCGN_1809, LCGN_1810, LCGN_1811, and LCGN_1812). Eight genes involved in pyruvate and carbohydrate metabolism and the citrate cycle (*glmS*, *glmM, galE*, PTS-Man-EIIC and EIID *pdhA, pdhB*, DLAT) were repressed by *S. aureus*. With low aeration, the enolase encoding gene was repressed by *S. aureus.*

Four biological processes related to transport functions were affected (**Figure 2D**): “transport,” “transmembrane transport,” “organic substance transport” and “ion transport.” In pure culture, genes involved in the transport of cobalt/zinc/cadmium (LCGN_1867) and an unspecified monosaccharide (LCGN_0332) were over-expressed with high aeration while a gene involved in copper transport (LCGN_1427) was repressed. Two genes of the *fur* regulon (ferrous iron transport) were over-expressed with high aeration, one of them in pure culture (*feoA*) and one in co-culture (*feoB*).

With low aeration, the H_2_O_2_ synthesis gene *sodA* was up-regulated by *S. aureus.* The H_2_O_2_ degradation gene *ahpC* was repressed by high aeration in co-culture but not in pure culture. In pure culture, two genes related to O_2_ consumption, noxE and LCGN_0208, were repressed and six genes related to ETC, *cydA*, *cydC, menB*, *menC*, *menD*, and LCGN_0364, were over-expressed.

### Impact of Aeration on the Expression of *L. garvieae* Genes Potentially Involved in the Antagonism against *S. aureus*

To complete the RNA-seq data obtained only at 9 h, we determined the expression of ten genes from the cultures previously used for RNA-seq analyses, at 6, 9, and 24 h by RT-qPCR. These genes, potentially involved in the antagonism mechanisms, were involved in O_2_ consumption (*noxE* and *lox*), H_2_O_2_ synthesis (*pox* and *sodA*), H_2_O_2_ degradation (*ahpC*, *ahpF*, *gpx*, and *trxB1*) and resistance to other peroxides (*ohrA* and *ohrR*).

The presence of *S. aureus* induced only slight changes in their expression (data not shown). With high aeration, *ahpC* and *pox* were over-expressed 2.4-fold and 2.0-fold in the presence of *S. aureus* at 6 h. With low aeration, *S. aureus* induced a 3.1-fold over-expression of *trxB1* at 9 h and a 3.3-fold repression of *gpx* at 24 h.

Conversely, RT-qPCR results showed major differences in H_2_O_2_-metabolism gene expression between the two aeration levels (**Table 2**). The expression of *ohrA* was strongly induced under high aeration conditions in both pure culture and co-culture at 9 h. While RNA sequencing identified *trxA2* as up-regulated with high aeration in both pure culture and co-culture at 9 h (Supplementary Table 4), RT-qPCR identified other H_2_O_2_-degradation genes (*ahpC*, *ahpF*, *gpx*, and *trxB1*) as repressed at 6, 9, or 24 h. The lactate mono-oxygenase gene *lox* seemed slightly repressed with high aeration but this modification was significant only at 9 h in co-culture. In pure culture with high aeration, the expression of *noxE* reached a peak at 6 h when it was 11.0 times higher than with low aeration. With low aeration, *noxE* expression gradually increased over time. No significant difference in the expression of H_2_O_2_-synthesis genes (*pox* and *sodA*) according to aeration level was observed.

**Table 2:** Effect of high aeration level on the expression of *L. garvieae* genes in pure culture and co-culture, as determined by RT-qPCR.

		In pure culture^a^			In co-culture^a^		
	Gene	6 h	9 h	24 h	6 h	9 h	24 h
Degradation of H_ 2_O_2_	*ahpC*		0.6^∗^		0.6^∗^	0.4^∗∗^	
	*ahpF*		0.3^∗∗^	NT		0.4^∗^	NT
	*gpx*			0.2^∗^			0.4^∗^
	*trxB1*			0.2^∗∗^			0.2^∗∗^
Degradation of other peroxides	*ohrA*		16.7^∗∗^	NT	7.0^∗^	20.2^∗^	NT
O_2_ consumption/H_2_O synthesis	*noxE*	11.0^∗∗^	0.4^∗^	0.1^∗^			
	*lox*			NT		0.4^∗∗^	NT

^a^*Indicated values, at 6, 9, or 24 h in pure culture or in co-culture, corresponds to the ratios of gene expressions with high aeration to gene expressions with low aeration. Only ratios that are significant according to the Newman–Keuls test are shown (^∗^p-value < 0.1, ^∗∗^p-value < 0.05). NT, not tested*.

## Discussion

This study aimed to improve understanding of the mechanisms underlying the antagonism of *L. garvieae* against *S. aureus*, especially as regards H_2_O_2_-related pathways.

The high aeration level and the resulting high H_2_O_2_ concentration were associated with stronger inhibition of *S. aureus*, confirming previous observations under the same conditions (Alomar et al., 2008a; Delbes-Paus et al., 2010; Delpech et al., 2015). It was already known that this inhibition is associated with a modulation of the expression of *S. aureus* virulence genes and a repression of the H_2_O_2_ response, stress response and cell division genes of *S. aureus* by *L. garvieae* and with high aeration (Delpech et al., 2015). However, the transcriptome adaptation of *L. garvieae* to prevailing aeration conditions during this interaction had not been explored until this study. It is known that difference in aeration is associated with drastic modifications of the *L. lactis* transcriptome (Pedersen et al., 2008; Dijkstra et al., 2014) and consequent metabolic adaptations (Jensen et al., 2001; Nordkvist et al., 2003; Pedersen et al., 2008; Dijkstra et al., 2014; Larsen et al., 2016a,b). In accordance with these findings, we found that different aeration levels were associated with significant differences in the *L. garvieae* transcriptome in biological processes related to fundamental growth functions, nutritional and metabolic adaptations and transport functions. Moreover, *L. garvieae* genes involved in fundamental growth functions were slightly repressed by *S. aureus* under both aeration levels suggesting an impact of *S. aureus* on *L*. *garvieae* metabolism. While *L. garvieae* is a catalase-negative bacterium, the main *S. aureus* enzyme involved in H_2_O_2_ dismutation is the O_2_-forming catalase KatA (Cosgrove et al., 2007). The *S. aureus* catalase may affect the transcriptomic response of *L. garvieae* to different aeration conditions by modulating O_2_ and H_2_O_2_ concentrations. It is known that the *katA* gene of *S. aureus* SA15 is repressed by *L. garvieae* in high aeration conditions but not in low aeration conditions (Delpech et al., 2015). This may explain why the presence of *S. aureus* (*via* its catalase production) modulated the *L. garvieae* transcriptome response under different aeration conditions. Indeed, with low aeration, the *L. garvieae* H_2_O_2_ synthesis gene *sodA* expression was slightly higher in presence of *S. aureus*. With high aeration, the main enzymes involved in O_2_ consumption of *L. lactis* (NADH-oxidase NoxE and ETC enzymes (Tachon et al., 2010), were repressed only in *L. garvieae* pure culture. The expression of the *L. garvieae noxE* reached a peak at 6 h (beginning of exponential growth) and then decreased until 24 h. This suggests that *L. garvieae* NoxE is the main enzyme responsible for O_2_ consumption at the beginning of exponential growth with high aeration, as observed for *L. lactis* NoxE (Lopez de Felipe and Hugenholtz, 2001; Tachon et al., 2010). The induction of most of the *L. garvieae* ETC genes (*cydA*, *cydB*, *cydC*, *menB*, *menC*, *menD*, *menH*, and LCGN_0364) under high aeration conditions at 9 h (beginning of stationary phase) suggested that ETC may be involved in O_2_ consumption by *L. garvieae* during the stationary phase, as already shown with *L. lactis* (Tachon et al., 2009, 2010).

Previous studies have showed that hydrogen peroxide production by *L. garvieae* depends on aeration level and plays a key role in *S. aureus* inhibition. As regards *S. aureus*, Delpech et al. (2015) suggested that the stronger inhibition with a high aeration level may be caused by the higher concentration of H_2_O_2_ associated with the *L. garvieae*-induced repression of *S. aureus* genes involved in the H_2_O_2_ response (*katA* and *sodA* at 6 and 9 h) and cell division (*mraZ*, *mraW* and potentially the *dcw* cluster). As regards *L. garvieae*, our RNA-sequencing and RT-qPCR analyses showed an overexpression of the H_2_O_2_-degradation genes *ahpC*, *ahpF*, *gpx*, and *trxB1* under low aeration conditions compared to high aeration, while the expression of H_2_O_2_ synthesis genes *pox* and *sodA* remained stable. The expression of the main H_2_O_2_ degradation genes of Gram-positive bacteria is generally induced more under aeration and in the presence of H_2_O_2_ (Mostertz et al., 2004; Pedersen et al., 2008). Also, H_2_O_2_ metabolism may be very different in *L. garvieae* compared to other Gram-positive bacteria. Although the difference in H_2_O_2_ concentration between the two aeration levels was probably primarily conditioned by the availability of O_2_, our transcriptome results suggest that it was also associated with a control of H_2_O_2_ degradation by *L. garvieae* rather than with a control of H_2_O_2_ synthesis. Since the AhpCF peroxy-redoxin system was repressed, the OhrAR system was probably essential for the resistance of *L. garvieae* to ROS (other than H_2_O_2_) under the high aeration conditions. The widespread organic hydroperoxide detoxifying system *ohrAR*, known to be over-expressed under high O_2_ conditions (Mongkolsuk et al., 1998; Fuangthong et al., 2001; Chuchue et al., 2006; Oh et al., 2007; Atichartpongkul et al., 2010; da Silva Neto et al., 2012; Clair et al., 2013), was consistently induced in *L. garvieae* at the high aeration level.

The fact that *S. aureus* population levels were lower in the stationary phase in H_2_O_2_-free supernatant from a co-culture of *S. aureus* and *L. garvieae* revealed the presence of a new molecule involved in this inhibition. This effector is extracellular, is produced by *L. garvieae* during its exponential growth phase and can reduce the population level of *S. aureus* during its stationary growth phase. In view of the results of our enzymatic treatments on supernatants, the inhibitory gap during the stationary phase was probably caused neither by hydrogen peroxide, nor by a protein, nor by a lipid, nor by a polysaccharide. The only putative bacteriocin identified in the *L. garvieae* N201 genome was homologous to garvieaecin Q (GarQ, data not shown), a class IId bacteriocin (Tosukhowong et al., 2012). Class IId bacteriocins are generally sensitive to protease treatments as stringent as the one we used in this study (Kuo et al., 2013; Song et al., 2014), suggesting that garvieacin Q is unlikely to be the effector we are seeking. This effector may instead be related to several genes identified by RNA seq as being regulated by aeration level, such as genes involved in metal homeostasis (e.g., siderophores (Hannauer et al., 2015), chemical and ionic equilibrium (Doyle et al., 1975; Chudobova et al., 2015), transport of vitamin-related compounds (Schlievert et al., 2013) and export of unknown proteins. For example, the differential expression of ferrous ion transport encoding genes has already been observed in *L. lactis* under oxidative stress in milk (Larsen et al., 2016b). It may also be related to signaling molecules (stress, quorum sensing). It is known that *L. garvieae* can modify the expression of several *S. aureus* genes involved in environment-sensing systems like the *agr* system, CodY or two-component systems SaeRS and SrrAB (Delpech et al., 2015).

RNA-seq revealed variations in the expression of the *codY* gene (involved in nutritional adaptation (Guédon et al., 2001; Ercan et al., 2015), and of several nutritional-related metabolisms (lysine, threonine, mannitol, aspartate, ribose, fructose, and galactose). This suggests that *L. garvieae* adapts its nutritional behavior to the prevailing aeration level and the presence or absence of *S. aureus*. In a rich medium like BHI, there should be little nutritional competition. It is known that *L. garvieae* can consume all the L-threonine in micro-filtered milk in less than 3 h (Alomar et al., 2008b) and that *S. aureus* growth could be inhibited by L-threonine depletion (Pohl et al., 2009). However, we showed that the antagonism of *L. garvieae* against *S. aureus* was not associated with nutritional competition for L-threonine in micro-filtered milk (see Supplementary Table 5).

## Conclusion

RNA sequencing analyses revealed a *L. garvieae* transcriptome adaptation to aeration level. This adaptation differed depending on the presence or absence of *S. aureus*. Our findings show that the control of autogenic H_2_O_2_ levels by *L. garvieae* was probably carried out by H_2_O_2_ degradation genes rather than H_2_O_2_ synthesis genes. Our study also leads us to suggest that an unidentified effector was involved in the inhibition of *S. aureus* in the stationary phase. The potential inhibitory role of metals, siderophores and signal molecules (e.g., stress signal, quorum sensing) generated by *L. garvieae* should be investigated. In order to promote the use of H_2_O_2_-producing bacteria as bio-preservation agents, future studies should explore the relation between H_2_O_2_-metabolism, H_2_O_2_-producing LAB and the pathogen they inhibit.

## Author Contributions

PD carried out all the experiments, excluding preparation of cDNA libraries and RNA sequencing, and drafted the manuscript helped by CD and SB. ER, ED, and GB analyzed RNA sequencing data. SN performed the RNA sequencing (preparation of libraries and the sequencing itself). GG, M-CM, CD and SB conceived the study. All authors read and approved the final manuscript.

## Conflict of Interest Statement

The authors declare that the research was conducted in the absence of any commercial or financial relationships that could be construed as a potential conflict of interest.

## Footnotes

1 http://www.bioinformatics.babraham.ac.uk/projects/fastqc/

2 http://www.R-project.org/

3 http://blast.ncbi.nlm.nih.gov/Blast.cgi
