# Supplementary material for: New Insights into the Anti-pathogenic Potential of Lactococcus garvieae against Staphylococcus aureus Based on RNA Sequencing Profiling
Source: Front Microbiol. 2017 Mar 8;8:359. doi: 10.3389/fmicb.2017.00359 (PMC5340753; doi:10.3389/fmicb.2017.00359)
Supplement: Supplementary file 1 [file Table_1.docx]

**Supplementary table 1. Number of reads generated by RNA-sequencing for each condition after bioinformatics and filtering treatments. ^(a)^ Tot = Total of reads generated on the *HiSeq 2000* (Illumina) after the filtering of image. ^(b)^ Map = reads mapped on the two reference genomes (Map = Tot - Multi - Unk). ^(c)^ Multi = reads which mapped on at least two different sites of the references genomes. ^(d)^ Unk = reads which did not map with any site of the reference genomes (unknown reads).**

| Samples | | | Number of reads | | | |
| --- | --- | --- | --- | --- | --- | --- |
| Culture | Aeration level | Replicate | Tot ^(a)^ | Map ^(b)^ | Multi ^(c)^ | Unk ^(d)^ |
| Pure culture | High | A | 15,441,932 | 14,049,750 | 48,851 | 1,343,331 |
| Pure culture | High | B | 14,435,199 | 12,935,269 | 55,060 | 1,444,870 |
| Pure culture | High | C | 12,365,133 | 10,824,067 | 104,424 | 1,436,642 |
| Pure culture | Low | A | 13,461,986 | 11,669,070 | 428,462 | 1,364,454 |
| Pure culture | Low | B | 13,282,584 | 11,622,174 | 72,065 | 1,588,345 |
| Pure culture | Low | C | 11,447,286 | 9,828,863 | 72,059 | 1,546,364 |
| Co-culture | High | A | 12,835,317 | 11,508,274 | 54,644 | 1,272,399 |
| Co-culture | High | B | 13,823,339 | 12,111,806 | 371,004 | 1,340,529 |
| Co-culture | High | C | 13,627,195 | 12,156,053 | 43,826 | 1,427,316 |
| Co-culture | Low | A | 14,219,669 | 12,788,703 | 44,121 | 1,386,845 |
| Co-culture | Low | B | 15,670,131 | 13,426,626 | 459,401 | 1,784,104 |
| Co-culture | Low | C | 14,721,283 | 12,231,362 | 212,832 | 2,277,089 |
