# Supplementary material for: New Insights into the Anti-pathogenic Potential of Lactococcus garvieae against Staphylococcus aureus Based on RNA Sequencing Profiling
Source: Front Microbiol. 2017 Mar 8;8:359. doi: 10.3389/fmicb.2017.00359 (PMC5340753; doi:10.3389/fmicb.2017.00359)
Supplement: Supplementary file 2 [file Table_2.docx]

**Supplementary table 2.** **Targeted *L. garvieae* genes primers for qPCR analysis.
^a^ reference gene.**

| Gene | Description | Forward primer | Reverse primer |
| --- | --- | --- | --- |
| *tufB* ^a^ | elongation factor Tu | CTGGTATCGAAATGTTCCGTAAAA | CCACGGAGAAGTACACCAACGT |
| *ahpC* | Alkyl hydroperoxidase C | GATAAAGTGCGTGCTGCACAAT | TTTCCGCATCTTCTTTCCATTT |
| *ahpF* | alkyl hydroperoxidase F | ATTGCTTATTGTCCGCATTGTG | CTCCCCCAACTACGGCAACT |
| *gpx* | glutathione peroxidase | GTACAACTTTTCCACGCTTTCAAA | CCAGCGGGCTTCCTTTTT |
| *lox* | L-lactate 2-monooxygenase | CAGGACGACCAATGGCAACT | TGTACGTCGTGGTGAGCATGT |
| *noxE* | NADH oxidase | CACGCTAACGGTGCTAAAAAAGT | CCGTGGTTAGTGTTGAAAACGATAGTT |
| *ohrA* | organic hydrogen peroxide resistance A | CATGGGCCATTTCTAACAATTCT | TGTTGACATCGTCGGACACA |
| *ohrR* | organic hydrogen peroxide resistance regulator | TGCTTTCAACTGTACATTGCTTCTAA | AACGCATTGACGGTCATGTC |
| *poxB* | pyruvate oxidase | TGGCGTACATCTGGCTTGTTT | AAGTCTAGTTTGGCCGAAATCG |
| *sodA* | superoxide dismutase | ACCACACAATGTTCTGGGAATG | TGCTACAGCGATATCACCTGTTG |
| *trxB1* | thioredoxine reductase | GCCTTTAGCTGATCTTGGTGT | TTGATTGTTTTTGTTGCACCATT |
