# Supplementary material for: New Insights into the Anti-pathogenic Potential of Lactococcus garvieae against Staphylococcus aureus Based on RNA Sequencing Profiling
Source: Front Microbiol. 2017 Mar 8;8:359. doi: 10.3389/fmicb.2017.00359 (PMC5340753; doi:10.3389/fmicb.2017.00359)
Supplement: Supplementary file 3 [file Table_3.docx]

**Supplementary table 3.** Genes differentially expressed in presence of *S. aureus* either under high aeration (A) or under low aeration (B) according to the regression analysis. Only significant ratios of the expression of genes are indicated.

**(A) Genes differentially expressed in presence of *S. aureus*, under high aeration.**

| **Name** | **Description** | **Ratio of gene expression in co-culture with high aeration** |
| --- | --- | --- |
| LCGN_1184 | putative_scaffolding_protein | 2,370787 |
| LytA | Cell_wall-associated_murein_hydrolase_LytA | 2,293478 |
| LCGN_1920 | 2-amino-3-ketobutyrate_coenzyme_A_ligase_(EC_2.3.1.29) | 1,250616 |
| PTS-Bgl-EIIB, bglF, bglP | PTS_system2C_beta-glucoside-specific_IIB_component_(EC_2.7.1.69)_/_PTS_system2C_beta-glucoside-specific_IIC_component_(EC_2.7.1.69)_/_PTS_system2C_beta-glucoside-specific_IIA_component_(EC_2.7.1.69) | 1,234019 |
| rpoA | DNA-directed_RNA_polymerase_alpha_subunit_(EC_2.7.7.6) | 1,229443 |
| pyrG | CTP_synthase_(EC_6.3.4.2) | 1,208749 |
| galE | L-threonine_3-dehydrogenase_(EC_1.1.1.103) | 1,196461 |
| LCGN_5 | Phosphoesterase2C_DHH_family_protein | 1,19205 |
| RP-S9 | SSU_ribosomal_protein_S9p_(S16e) | 1,177668 |
| guaB | Inosine-5'-monophosphate_dehydrogenase_(EC_1.1.1.205) | 1,175888 |
| secY | Preprotein_translocase_secY_subunit_(TC_3.A.5.1.1) | 1,166813 |
| LCGN_60 | oligopeptide_transporter2C_OPT_family | 1,154394 |
| glyA | Serine_hydroxymethyltransferase_(EC_2.1.2.1) | 1,148323 |
| tig | Cell_division_trigger_factor_(EC_5.2.1.8) | 1,146252 |
|  | Acetolactate_synthase2C_catabolic_(EC_2.2.1.6) | 1,142785 |
| sufS | Cysteine_desulfurase_(EC_2.8.1.7)2C_SufS_subfamily | 1,140339 |
| PDHB | Acetoin_dehydrogenase_E1_component_beta-subunit_(EC_1.2.4.-) | 1,133952 |
| fhs | Formate--tetrahydrofolate_ligase_(EC_6.3.4.3) | 1,130159 |
| PTS-Man-EIIC | PTS_system2C_mannose-specific_IIC_component_(EC_2.7.1.69) | 1,124961 |
| DLAT | Dihydrolipoamide_acetyltransferase_component_(E2)_of_acetoin_dehydrogenase_complex_(EC_2.3.1.-) | 1,121964 |
| aatA | Aspartate_aminotransferase_(EC_2.6.1.1) | 1,120709 |
| glmM | Phosphoglucomutase_(EC_5.4.2.2) | 1,117149 |
| lplA | Lipoate-protein_ligase_A | 1,115855 |
| ftsH | Cell_division_protein_FtsH_(EC_3.4.24.-) | 1,108413 |
| PDHA | Acetoin_dehydrogenase_E1_component_alpha-subunit_(EC_1.2.4.-) | 1,107071 |
| glmS | Glucosamine--fructose-6-phosphate_aminotransferase_[isomerizing]_(EC_2.6.1.16) | 1,097871 |
| PTS-Man-EIID | PTS_system2C_mannose-specific_IID_component_(EC_2.7.1.69) | 1,091906 |
| mntH | Manganese_transport_protein_MntH | 1,082766 |
| sufB | Iron-sulfur_cluster_assembly_protein_SufB | 1,080058 |
| mgtA/mgtB | Mg(2+)_transport_ATPase2C_P-type_(EC_3.6.3.2) | 1,079033 |
| ftsZ | Cell_division_protein_FtsZ_(EC_3.4.24.-) | 1,072678 |
| arcA | Arginine_deiminase_(EC_3.5.3.6) | 1,055522 |
| ald | Alanine_dehydrogenase_(EC_1.4.1.1) | 1,055497 |
| pepT | Tripeptide_aminopeptidase_(EC_3.4.11.4) | 1,055433 |
| LCGN_146 | Putative_Dihydrolipoamide_dehydrogenase_(EC_1.8.1.4)%3B_Mercuric_ion_reductase_(EC_1.16.1.1)%3B_PF00070_family2C_FAD-dependent_NAD(P)-disulphide_oxidoreductase | 1,049501 |
| GbpB/SagA/PcsB | Secreted_antigen_GbpB/SagA/PcsB2C_putative_peptidoglycan_hydrolase | 1,042212 |
| OTC | Ornithine_carbamoyltransferase_(EC_2.1.3.3) | 1,002308 |

1. **Genes differentially expressed in presence of *S. aureus*, under low aeration.**

| **Name** | **Description** | **Ratio of gene expression in co-culture with low aeration** |
| --- | --- | --- |
| eno | Enolase_(EC_4.2.1.11) | 1,027801 |
| LCGN_144 | Transcriptional_regulator2C_TetR_family | 1,012251 |
| sod | Manganese_superoxide_dismutase_(EC_1.15.1.1) | 0,981607 |
| LCGN_1087 | ankyrin_repeat_family_protein | 0,885318 |
| nrd | Ribonucleotide_reductase_of_class_Ib_(aerobic)2C_alpha_subunit_(EC_1.17.4.1) | 0,772673 |
