# Supplementary material for: New Insights into the Anti-pathogenic Potential of Lactococcus garvieae against Staphylococcus aureus Based on RNA Sequencing Profiling
Source: Front Microbiol. 2017 Mar 8;8:359. doi: 10.3389/fmicb.2017.00359 (PMC5340753; doi:10.3389/fmicb.2017.00359)
Supplement: Supplementary file 4 [file Table_4.docx]

**Supplementary table 4.** Genes differentially expressed with different aeration levels (high and low) according to the three R packages used (DESeq, DESeq2 and EdgeR). Only significant ratios of the expression of genes with high aeration / with low aeration are indicated either in pure culture or in co-culture.

1. Genes differentially expressed and discussed in this article.
2. Other genes differentially expressed both in pure culture and in co-culture.
3. Other genes differentially expressed exclusively in pure culture.
4. Other genes differentially expressed exclusively in co-culture.

**(A) Genes differentially expressed and discussed in this article.**

| Name | Description | ratio of gene expression in pure culture with high aeration / with low aeration | | | ratio of gene expression in co-culture with high aeration / with low aeration | | |
| --- | --- | --- | --- | --- | --- | --- | --- |
|  |  | DESeq | DESeq2 | EdgeR | DESeq | DESeq2 | EdgeR |
| **O_2_ metabolism** | |  |  |  |  |  |  |
|  | **Electron Transport Chain** |  |  |  |  |  |  |
| *cydB* | Cytochrome d ubiquinol oxidase subunit II |  | 1.96 | 2.72 | 2.35 | 2.27 | 2.78 |
| *menH* | 2-succinyl-6-hydroxy-2,4-cyclohexadiene-1-carboxylate synthase |  | 2.67 |  |  | 2.64 |  |
| *ubiE* | Ubiquinone/menaquinone biosynthesis methyltransferase UBIE | 0.13 | 0.15 | 0.18 | 0.17 | 0.18 | 0.20 |
| *cydA* | Cytochrome d ubiquinol oxidase subunit I |  |  | 2.14 |  |  |  |
| *cydC* | Transport ATP-binding protein CydC |  |  | 2.19 |  |  |  |
| *menC* | O-succinylbenzoate-CoA synthase |  | 2.56 |  |  |  |  |
| *menB* | Naphthoate synthase |  | 2.23 | 2.87 |  |  |  |
| *menD* | 2-succinyl-5-enolpyruvyl-6-hydroxy-3-cyclohexene-1-carboxylic-acid synthase |  | 2.73 | 3.50 |  |  |  |
| LCGN_0364 | Putative coproporphyrinogen III oxidase of BS HemN-type oxygen-independent in heat shock gene cluster |  | 1.99 |  |  |  |  |
|  | **Other** |  |  |  |  |  |  |
| *lox* | Lactate 2-monooxygenase |  | 0.26 |  |  | 0.46 |  |
| *noxE* | NADH oxidase |  | 0.30 | 0.34 |  |  |  |
| LCGN_0208 | Lactate 2-monooxygenase |  | 0.31 |  |  |  |  |
| **Peroxide / H_2_O_2_ metabolism** | |  |  |  |  |  |  |
|  | **Synthesis of H_2_O_2_** |  |  |  |  |  |  |
| *pox* | Pyruvate oxidase |  | 0.40 |  |  | 0.50 |  |
| *spxA1* | Transcriptional regulator SpxA1 |  | 0.14 | 0.14 |  | 0.20 | 0.22 |
|  | **Resistance to H_2_O_2_** |  |  |  |  |  |  |
| *trxA2* | Thioredoxin |  | 2.22 | 2.90 | 2.50 | 2.43 | 2.91 |
| *ahpF* | Alkyl hydroperoxide reductase protein F |  | 0.35 | 0.44 |  | 0.42 |  |
| *ahpC* | Alkyl hydroperoxide reductase protein C |  |  |  |  | 0.53 |  |
|  | **Resistance to other peroxides** |  |  |  |  |  |  |
| *ohrA* | Organic hydroperoxide resistance protein | 33.80 | 24.43 | 48.30 | 45.26 | 39.96 | 55.21 |
| *ohrR* | Organic hydroperoxide resistance transcriptional regulator |  | 3.25 |  |  |  | 4.64 |
| **Nutritional metabolisms** | |  |  |  |  |  |  |
|  | **Threonine metabolism** |  |  |  |  |  |  |
| LCGN_1919 | L-threonine 3-dehydrogenase |  | 2.54 | 3.19 | 2.48 | 2.41 | 2.90 |
| LCGN_1920 | 2-amino-3-ketobutyrate coenzyme A ligase |  | 2.82 | 3.62 | 2.65 | 2.59 | 3.10 |
| *ilvA* | Threonine dehydratase, catabolic |  | 0.22 | 0.26 |  | 0.25 | 0.30 |
| LCGN_1922 | Alanine dehydrogenase |  | 0.28 | 0.35 |  | 0.38 |  |
|  | **Threonine and lysine metabolism** |  |  |  |  |  |  |
| LCGN_0576 | Aspartate-semialdehyde dehydrogenase |  | 3.05 |  |  |  |  |
| LCGN_0577 | Aspartokinase |  | 3.20 |  |  |  |  |
|  | **Lysine metabolism** |  |  |  |  |  |  |
| LCGN_0575 | 2345-tetrahydropyridine-26-dicarboxylate N-acetyltransferase |  | 3.06 |  |  |  |  |
| LCGN_0578 | N-acetyl-LL-diaminopimelate deacetylase |  | 2.54 |  |  |  |  |
|  | **Galactose metabolism** |  |  |  |  |  |  |
| LCGN_1809 | UDP-glucose 4-epimerase |  |  |  |  | 2.89 | 3.38 |
| LCGN_1810 | Galactose-1-phosphate uridylyltransferase |  |  |  |  | 4.06 | 5.33 |
| LCGN_1811 | Galactokinase |  |  |  |  | 4.43 | 5.92 |
| LCGN_1812 | Aldose 1-epimerase |  |  |  | 6.05 | 4.87 | 6.34 |
| **Transport and extracellular compounds** | |  |  |  |  |  |  |
|  | **Bacteriocin** |  |  |  |  |  |  |
| LCGN_0476 | putative garvieaecin Q |  | 0.34 |  |  | 0.41 |  |
|  | **Vitamins and vitamers** |  |  |  |  |  |  |
| LCGN_0251 | Substrate-specific component RibU of riboflavin ECF transporter |  | 0.13 | 0.13 |  | 0.12 | 0.11 |
| LCGN_0714 | 5-formyltetrahydrofolate cyclo-ligase |  | 2.91 |  | 3.36 | 3.26 | 3.70 |
| LCGN_1870 | Substrate-specific component FolT of folate ECF transporter | 4.97 | 4.16 | 5.62 | 3.59 | 3.27 |  |
|  | **Monosaccharide** |  |  |  |  |  |  |
| LCGN_0332 | Unspecified monosaccharide ABC transport system permease component 2 |  | 2.01 |  |  |  |  |
|  | **Metals** |  |  |  |  |  |  |
| *znuA* | Zinc ABC transporter, periplasmic-binding protein ZnuA |  | 0.25 |  |  | 0.35 |  |
| LCGN_0683 | Lead, cadmium, zinc and mercury transporting ATPase %3B Copper-translocating P-type ATPase | 0.15 | 0.17 | 0.20 | 0.19 | 0.20 | 0.22 |
| LCGN_1104 | Lead, cadmium, zinc and mercury transporting ATPase %3B Copper-translocating P-type ATPase |  |  | 2.82 |  | 2.12 | 2.59 |
| LCGN_0139 | Lead cadmium zinc a mercury transporting ATPase %3B Copper-translocating P-type ATPase |  | 0.32 |  |  |  |  |
| LCGN_1428 | Lead cadmium zinc a mercury transporting ATPase %3B Copper-translocating P-type ATPase |  | 0.31 |  |  |  |  |
| LCGN_1867 | Cobalt-zinc-cadmium resistance protein |  | 2.12 |  |  |  |  |
| LCGN_1426 | Negative transcriptional regulator-copper transport operon |  | 0.39 |  |  |  |  |
| LCGN_1427 | Copper chaperone |  | 0.20 | 0.23 |  |  |  |
| *feoA* | Ferrous iron transport protein A |  | 0.33 |  |  |  |  |
| *feoB* | Ferrous iron transport protein B |  |  |  |  | 0.32 |  |
| **Stress response** | |  |  |  |  |  |  |
| *hrcA* | Heat-inducible transcription repressor HrcA | 0.06 | 0.08 | 0.08 | 0.19 | 0.22 | 0.22 |
| *groES* | Heat shock protein 60 family co-chaperone GroES |  | 0.35 | 0.44 |  | 0.47 |  |
| *groEL* | Heat shock protein 60 family chaperone GroEL |  | 0.23 | 0.27 | 0.31 | 0.33 | 0.38 |
| *dnaK* | Chaperone protein DnaK | 0.04 | 0.07 | 0.06 | 0.07 | 0.08 | 0.09 |
| *dnaJ* | Chaperone protein DnaJ | 0.11 | 0.15 | 0.16 | 0.23 | 0.24 | 0.27 |
| *grpE* | Heat shock protein GrpE | 0.04 | 0.05 | 0.05 | 0.12 | 0.13 | 0.14 |
| *clpB* | ClpB protein | 0.12 | 0.14 | 0.17 | 0.23 | 0.24 | 0.27 |
| LCGN_1088 | Universal stress protein family |  | 0.31 | 0.37 |  | 0.40 |  |
| LCGN_1532 | Universal stress protein family |  | 0.26 | 0.32 | 0.32 | 0.34 | 0.38 |
| LCGN_1556 | Universal stress protein family | 0.13 | 0.15 | 0.18 |  | 0.21 | 0.23 |
| LCGN_1557 | Universal stress protein family |  | 0.45 |  |  | 0.47 |  |
| *uspA* | Universal stress protein UspA |  | 0.40 |  |  | 0.45 |  |

1. **Other genes differentially expressed both in pure culture and in co-culture.**

| Name | Description | ratio of gene expression in pure culture with high aeration / with low aeration | | | ratio of gene expression in co-culture with high aeration / with low aeration | | |
| --- | --- | --- | --- | --- | --- | --- | --- |
|  |  | DESeq | DESeq2 | EdgeR | DESeq | DESeq2 | EdgeR |
| *LCGN_0004* | Predicted sugar phosphatase of HAD family |  | 0.30 | 0.37 |  | 0.30 | 0.34 |
| *LCGN_0006* | LSU ribosomal protein L9p |  | 0.40 |  |  | 0.40 |  |
| *LCGN_0059* | hypothetical protein | 0.18 | 0.20 | 0.23 | 0.29 | 0.30 | 0.34 |
| *LCGN_090* | COG3525: N-acetyl-beta-hexosaminidase |  | 0.34 |  |  | 0.32 |  |
| *LCGN_0116* | rRNA methylase |  | 0.38 |  |  | 0.48 |  |
| *LCGN_0117* | Acylphosphatephosphohydrolase, putative |  | 0.52 |  |  | 0.50 |  |
| *ptbA* | PTS system, N-acetylglucosamine-specific IIA component / PTS system, N-acetylglucosamine-specific IIB component / PTS system, N-acetylglucosamine-specific IIC component |  | 1.97 |  |  | 2.29 |  |
| *LCGN_0140* | Transcriptional regulator, PadR family |  | 3.61 |  |  | 4.32 | 5.44 |
| *LCGN_0141* | similar to membrane protein |  | 3.24 |  |  | 3.86 | 4.78 |
| *LCGN_0142* | hypothetical protein |  | 3.15 | 4.21 | 4.58 | 4.24 | 4.92 |
| *bacA / uppP* | Undecaprenyl-diphosphatase |  | 3.29 | 4.26 | 3.57 | 3.42 | 3.89 |
| *LCGN_0144* | Transcriptional regulator, TetR family |  | 2.67 | 4.48 | 3.13 |  | 3.98 |
| *LCGN_0145* | hypothetical protein | 4.76 | 3.87 | 7.13 | 4.85 | 4.19 | 6.08 |
| *LCGN_0146* | Putative Dihydrolipoamide dehydrogenase %3B Mercuric ion reductase %3B PF00070 family, FAD-dependent NAD(P)-disulphide oxidoreductase | 7.38 | 5.84 | 10.86 | 9.94 | 8.56 | 12.04 |
| *cblB / metC* | Cystathionine gamma-lyase | 8.51 | 7.11 | 11.92 | 9.48 | 8.49 | 11.51 |
| *LCGN_0148* | hypothetical protein |  | 0.49 |  |  | 0.45 |  |
| *LCGN_0149* | hypothetical protein |  | 0.43 |  | 0.37 | 0.39 |  |
| *LCGN_0153* | FMN-dependent NADH-azoreductase | 0.22 | 0.24 |  |  | 0.30 |  |
| *LCGN_0154* | Ribosomal subunit interface protein |  | 0.28 |  | 0.34 | 0.35 |  |
| *LCGN_0158* | Glycine betaine ABC transport system, permease protein OpuAB / Glycine betaine ABC transport system, glycine betaine-binding protein OpuAC |  | 0.40 |  | 0.36 | 0.38 |  |
| *LCGN_0199* | Ribose ABC transport system, high affinity permeaseRbsD |  | 3.15 |  |  | 2.93 |  |
| *LCGN_0200* | Ribokinase |  | 3.67 |  | 3.44 | 3.17 |  |
| *rbsR* | Ribose operon repressor |  | 3.51 |  | 3.58 | 3.29 |  |
| *LCGN_0203* | hypothetical protein |  | 0.42 |  |  | 0.51 |  |
| *LCGN_0204* | hypothetical protein |  | 0.40 |  |  | 0.50 |  |
| *LCGN_0233* | multidrug resistance protein |  | 0.43 |  |  | 0.51 |  |
| *LCGN_0244* | hypothetical protein |  | 0.27 | 0.33 | 0.32 | 0.34 | 0.38 |
| *LCGN_0276* | Dihydroorotase | 0.13 | 0.16 | 0.15 |  | 0.21 | 0.20 |
| *LCGN_0277* | Orotatephosphoribosyltransferase |  | 0.16 | 0.15 | 0.25 | 0.27 |  |
| *LCGN_0287* | hypothetical protein |  | 0.30 | 0.34 |  | 0.40 |  |
| *vraS* | Sensor histidine kinase VraS |  | 0.40 |  |  | 0.47 |  |
| *vraR* | Two component transcriptional regulator VraR |  | 0.46 |  |  | 0.52 |  |
| *LCGN_0295* | hypothetical protein |  | 0.29 | 0.36 | 0.32 | 0.34 | 0.38 |
| *LCGN_0300* | Carbamoyl-phosphate synthase large chain |  | 0.25 | 0.22 |  | 0.21 | 0.18 |
| *pydB* | Dihydroorotate dehydrogenase electron transfer subunit B |  | 0.17 | 0.10 |  | 0.17 | 0.12 |
| *elaA* | ElaA protein |  | 2.15 |  |  | 1.89 |  |
| *parC* | Topoisomerase IV subunit A |  | 2.55 | 3.32 |  | 2.21 | 2.43 |
| *LCGN_0365* | Phage lysin, glycosyl hydrolase, family 25 |  | 2.66 |  |  | 2.17 |  |
| *LCGN_0368* | Transporter |  | 4.40 | 6.13 | 4.48 | 4.11 | 4.69 |
| *LCGN_0398* | putative amino acid permease | 0.03 | 0.05 | 0.04 | 0.04 | 0.04 | 0.04 |
| *LCGN_0402* | hypothetical protein |  | 0.31 | 0.38 |  | 0.31 | 0.35 |
| *LCGN_0411* | transcription regulator |  | 0.27 | 0.32 |  | 0.27 | 0.31 |
| *LCGN_0412* | hypothetical protein |  | 0.32 |  |  | 0.31 |  |
| *LCGN_0420* | Transcriptional regulator, TetR family |  | 0.38 |  |  | 0.21 |  |
| *LCGN_0454* | Glycerol kinase | 0.06 | 0.07 | 0.07 | 0.04 | 0.04 | 0.05 |
| *LCGN_0455* | Aerobic glycerol-3-phosphate dehydrogenase | 0.10 | 0.11 | 0.12 | 0.07 | 0.08 | 0.08 |
| *glpF* | Glycerol uptake facilitator protein |  | 0.28 | 0.29 |  | 0.18 | 0.17 |
| *LCGN_0483* | putative acetyltransferase |  | 2.23 | 3.01 |  | 1.81 | 2.20 |
| *LCGN_0494* | hypothetical protein |  | 0.42 |  |  | 0.46 |  |
| *LCGN_0513* | PTS system N-acetylgalactosamine-specific IIA component | 0.06 | 0.09 | 0.08 | 0.09 |  | 0.10 |
| *ptnD* | PTS system, mannose-specific IID component | 0.10 | 0.14 | 0.13 | 0.10 |  | 0.12 |
| *LCGN_0522* | Galactosamine-6-phosphate isomerase | 0.22 | 0.24 |  | 0.22 | 0.23 |  |
| *LCGN_0523* | Tagatose-6-phosphate kinase | 0.22 | 0.24 |  | 0.25 | 0.26 |  |
| *lacD / lacF* | Tagatose 1,6-diphosphate aldolase |  | 0.25 |  | 0.25 | 0.26 |  |
| *LCGN_0555* | UDP-N-acetylglucosamine 2-epimerase |  | 0.45 |  |  | 0.50 |  |
| *LCGN_0564* | conserved hypothetical protein | 6.27 | 4.50 | 6.67 | 6.93 | 5.98 | 7.49 |
| *LCGN_0565* | Transcriptional regulator, MarR family | 7.53 | 5.35 | 8.12 | 8.05 | 6.65 | 8.53 |
| *LCGN_0566* | CAAX amino terminal protease family | 8.23 | 5.47 | 9.10 |  | 5.55 | 7.70 |
| *LCGN_0567* | putative tryptophan transport protein | 10.36 | 6.20 | 10.93 | 10.74 | 7.49 | 11.05 |
| *LCGN_0610* | hypothetical protein | 34.11 | 25.29 | 48.32 | 42.40 | 36.50 | 52.69 |
| *LCGN_0625* | Alpha-acetolactate decarboxylase |  | 2.34 | 3.08 | 3.00 | 2.91 | 3.46 |
| *LCGN_0638* | hypothetical protein |  | 0.20 |  | 0.21 | 0.22 | 0.23 |
| *LCGN_0660* | Dihydrolipoamide dehydrogenase of acetoin dehydrogenase |  | 2.79 | 3.88 | 2.57 | 2.46 | 3.14 |
| *LCGN_0661* | Dihydrolipoamide acetyltransferase component (E2) of acetoin dehydrogenase complex |  |  | 2.95 |  | 2.05 | 2.63 |
| *LCGN_0699* | Ribonuclease P protein component |  | 2.50 |  |  | 2.39 |  |
| *gatA* | Aspartyl-tRNA(Asn) amidotransferase subunit A @ Glutamyl-tRNA(Gln) amidotransferase subunit A |  | 1.92 | 2.55 |  | 1.95 | 2.24 |
| *gatB* | Aspartyl-tRNA(Asn) amidotransferase subunit B @ Glutamyl-tRNA(Gln) amidotransferase subunit B |  | 2.18 | 2.84 |  | 2.22 | 2.55 |
| *glpG* | GlpG protein (membrane protein of glp regulon) |  | 3.11 |  | 3.19 | 3.07 | 3.45 |
| *arcA* | Arginine deiminase | 11.61 | 9.59 | 13.51 | 9.75 | 9.44 | 11.03 |
| *arcB* | Ornithine carbamoyltransferase | 9.48 | 8.08 | 11.16 | 8.09 | 7.75 | 9.14 |
| *arcD* | Arginine/ornithine antiporter ArcD | 7.68 | 6.99 | 9.64 | 6.76 | 6.42 | 7.38 |
| *arcC* | Carbamate kinase | 7.98 | 7.14 | 9.84 | 6.19 | 5.92 | 6.95 |
| *LCGN_0724* | COG3760, similarity to aminoacyl-tRNA editing enzymes YbaK, ProX | 11.45 | 7.93 | 12.39 | 6.61 | 5.71 | 7.62 |
| *aspB* | Aspartate aminotransferase | 9.94 | 6.89 | 10.59 | 6.49 | 5.57 | 7.34 |
| *LCGN_0775* | Predicted L-lactate dehydrogenase, hypothetical protein subunit YkgG | 0.07 | 0.09 | 0.09 | 0.09 | 0.09 | 0.10 |
| *LCGN_0776* | Predicted L-lactate dehydrogenase, Iron-sulfur cluster-binding subunit YkgF |  | 0.23 | 0.21 |  | 0.22 | 0.21 |
| *LCGN_0777* | Predicted L-lactate dehydrogenase, Fe-S oxidoreductase subunit YkgE |  | 0.20 | 0.19 |  | 0.21 | 0.21 |
| *LCGN_0824* | Cysteine ABC transporter, substrate-binding protein |  |  | 2.10 |  | 1.96 | 2.39 |
| *LCGN_0842* | morphogenesis protein |  | 0.27 |  |  | 0.10 | 0.07 |
| *nifJ* | Pyruvate-flavodoxin oxidoreductase |  | 0.38 |  |  | 0.32 | 0.32 |
| *LCGN_0867* | Hypothetical protein ywlG |  | 0.46 |  |  | 0.51 |  |
| *LCGN_0885* | hypothetical protein |  | 0.27 |  |  | 0.28 |  |
| *LCGN_0903* | Ribosomal subunit interface protein | 0.23 | 0.24 | 0.29 |  | 0.27 | 0.30 |
| *LCGN_0913* | Pyruvate carboxyl transferase |  | 2.53 | 3.22 | 2.57 | 2.45 | 2.79 |
| *LCGN_0916* | hypothetical protein |  | 0.21 | 0.24 |  | 0.31 | 0.36 |
| *LCGN_0932* | Tripeptide aminopeptidase |  |  | 2.43 |  | 2.15 | 2.63 |
| *LCGN_0934* | hypothetical protein |  | 0.34 |  |  | 0.27 |  |
| *LCGN_0935* | hypothetical protein |  | 0.30 |  | 0.22 | 0.23 | 0.26 |
| *LCGN_0970* | Hypothetical, related to broad specificity phosphatases COG0406 |  | 0.38 |  |  | 0.47 |  |
| *spxA2* | Transcriptional regulator SpxA2 |  | 0.11 | 0.12 |  | 0.12 | 0.13 |
| *LCGN_0992* | Shikimate/quinate 5-dehydrogenase I beta |  | 0.32 |  | 0.39 | 0.40 |  |
| *LCGN_1010* | HD family hydrolase |  | 0.18 | 0.21 |  | 0.16 | 0.17 |
| *LCGN_1014* | membrane protein |  | 0.31 | 0.36 |  | 0.44 | 0.51 |
| *LCGN_1017* | hypothetical protein |  | 0.36 |  |  | 0.50 |  |
| *alaS* | Alanyl-tRNAsynthetase |  | 2.71 | 3.48 |  | 2.38 | 2.70 |
| *smpB* | tmRNA-binding protein SmpB |  | 0.31 |  |  | 0.37 |  |
| *serS* | Seryl-tRNAsynthetase |  | 0.22 | 0.26 |  | 0.21 | 0.23 |
| *malR* | Maltose operon transcriptional repressor MalR, LacI family |  | 0.44 |  |  | 0.51 |  |
| *mutY* | A/G-specific adenine glycosylase |  | 2.16 |  |  | 1.80 |  |
| *LCGN_1215* | Transcriptional regulator, TetR family | 0.18 | 0.21 | 0.23 |  | 0.29 | 0.32 |
| *LCGN_1391* | COG0779: clustered with transcription termination protein NusA |  | 0.35 |  | 0.39 | 0.40 |  |
| *nusA* | Transcription termination protein NusA |  | 0.35 | 0.44 |  | 0.42 |  |
| *LCGN_1393* | COG2740: Predicted nucleic-acid-binding protein implicated in transcription termination |  | 0.37 |  |  | 0.40 |  |
| *LCGN_1394* | ribosomal protein L7Ae family protein |  | 0.32 |  |  | 0.40 |  |
| *infB* | Translation initiation factor 2 |  | 0.27 | 0.34 |  | 0.32 | 0.37 |
| *rbfA* | Ribosome-binding factor A | 0.18 | 0.21 | 0.25 |  | 0.28 | 0.33 |
| *celB* | PTS system, cellobiose-specific IIC component |  | 2.69 |  |  | 2.38 |  |
| *LCGN_1432* | hypothetical protein |  | 2.87 |  |  | 2.18 |  |
| *LCGN_1433* | Acetylornithine deacetylase/Succinyl-diaminopimelatedesuccinylase and related deacylases |  | 2.74 |  |  | 2.34 |  |
| *LCGN_1444* | 6-phosphogluconate dehydrogenase, decarboxylating |  | 0.27 |  |  | 0.33 |  |
| *LCGN_1445* | Gluconokinase |  | 0.43 |  | 0.36 | 0.38 |  |
| *pyrR* | Uracil phosphoribosyltransferase / Pyrimidine operon regulatory protein PyrR |  | 0.25 |  |  | 0.31 |  |
| *pyrP* | Uracil permease |  | 0.28 |  |  | 0.28 |  |
| *LCGN_1451* | Carbamoyl-phosphate synthase small chain |  | 0.21 | 0.14 |  |  | 0.16 |
| *LCGN_1453* | negative regulator of proteolysis |  | 0.27 |  |  |  | 0.24 |
| *LCGN_1471* | Glycerol kinase |  | 0.37 |  | 0.38 | 0.39 |  |
| *LCGN_1473* | Activator of (R)-2-hydroxyglutaryl-CoA dehydratase |  | 0.30 |  | 0.17 | 0.19 | 0.18 |
| *LCGN_1474* | Oxidoreductase |  | 0.30 |  | 0.16 |  | 0.18 |
| *LCGN_1481* | SSU ribosomal protein S16p |  | 0.43 |  |  | 0.54 |  |
| *LCGN_1487* | Fumarate reductase flavoprotein subunit | 0.08 | 0.09 | 0.10 | 0.14 | 0.16 | 0.17 |
| *LCGN_1504* | Arginine/ornithine antiporter ArcD | 0.15 | 0.17 | 0.19 | 0.16 | 0.16 | 0.18 |
| *LCGN_1505* | Xaa-His dipeptidase | 0.10 | 0.12 | 0.13 | 0.09 | 0.10 | 0.11 |
| *LCGN_1525* | 5'-nucleotidase YjjG |  |  | 2.57 |  | 1.97 |  |
| *LCGN_1530* | Glyoxalase family protein |  | 0.18 | 0.19 |  | 0.43 |  |
| *LCGN_1541* | Nucleoside-binding protein |  | 0.34 | 0.43 |  | 0.38 | 0.43 |
| *LCGN_1555* | hypothetical protein |  | 0.35 |  |  | 0.46 |  |
| *scpB* | Segregation and condensation protein B |  | 2.77 |  | 2.63 | 2.50 |  |
| *LCGN_1610* | Redox-sensitive transcriptional regulator (AT-rich DNA-binding protein) |  | 0.33 |  | 0.39 | 0.41 |  |
| *LCGN_1611* | Malolactic regulator | 5.11 | 4.55 | 6.29 | 4.61 | 4.40 | 5.17 |
| *LCGN_1625* | hypothetical protein |  | 2.47 | 3.12 |  | 2.19 |  |
| *LCGN_1626* | TPR-repeat-containing protein |  | 2.36 | 2.98 |  | 2.17 |  |
| *LCGN_1656* | PTS system, beta-glucoside-specific IIA/B/C component |  | 3.02 | 5.42 |  |  | 3.80 |
| *LCGN_1657* | 6-phospho-beta-glucosidase |  | 3.95 | 7.50 |  |  | 4.94 |
| *LCGN_1658* | 6-phospho-beta-glucosidase |  | 4.09 | 7.36 |  | 3.15 | 5.34 |
| *gidA* | tRNA uridine 5-carboxymethylaminomethyl modification enzyme GidA | 4.93 | 4.30 | 5.97 | 3.14 | 2.99 |  |
| *LCGN_1710* | cation-transporting ATPase |  | 0.38 |  | 0.30 | 0.32 |  |
| *LCGN_1736* | arginine repressor |  | 0.42 |  |  | 0.48 |  |
| *LCGN_1737* | hypothetical protein |  | 0.31 |  |  | 0.47 |  |
| *LCGN_1745* | Transcriptional regulator, repressor of the glutamine synthetase, MerR family |  | 0.33 |  |  | 0.49 |  |
| *LCGN_1766* | Fibronectin-binding protein |  | 2.00 | 2.56 |  | 2.01 |  |
| *adhE* | Alcohol dehydrogenase %3B Acetaldehyde dehydrogenase |  | 0.21 | 0.24 | 0.15 | 0.17 | 0.18 |
| *LCGN_1773* | 6-phosphogluconolactonase |  | 0.40 |  |  | 0.48 |  |
| *LCGN_1795* | tRNA dihydrouridine synthase B |  | 2.42 |  |  | 2.32 |  |
| *cutC* | Cytoplasmic copper homeostasis protein cutC |  | 0.35 |  |  | 0.46 |  |
| *LCGN_1853* | hypothetical protein |  | 0.37 | 0.48 |  | 0.52 |  |
| *LCGN_1890* | Orf32 |  | 0.35 |  |  | 0.48 |  |
| *nrdD* | Ribonucleotide reductase of class III (anaerobic), large subunit |  | 0.29 | 0.36 | 0.35 | 0.36 | 0.40 |
| *LCGN_1932* | LSU m5C1962 methyltransferase RlmI |  | 0.42 |  |  | 0.43 |  |

1. **Other genes differentially expressed exclusively in pure culture.**

| Name | Description | ratio of gene expression in pure culture with high aeration / with low aeration | | |
| --- | --- | --- | --- | --- |
|  |  | DESeq | DESeq2 | EdgeR |
| *LCGN_0078* | hypothetical protein |  | 0.48 |  |
| *LCGN_0091* | Putative stomatin/prohibitin-family membrane protease subunit YbbK |  | 0.32 | 0.41 |
| *LCGN_0097* | hypothetical protein |  | 0.42 |  |
| *LCGN_0105* | hypothetical protein |  | 0.46 |  |
| *LCGN_0163* | hypothetical protein |  | 0.29 |  |
| *LCGN_0170* | lmo0472 |  | 0.43 |  |
| *LCGN_0174* | hypothetical protein |  | 0.40 |  |
| *LCGN_0198* | Putative ribose uptake protein RbsU GRP transporter family |  | 3.51 |  |
| *LCGN_0209* | Conserved hypothetical protein ArsC related |  | 0.33 |  |
| *LCGN_0228* | Nucleoside diphosphate kinase |  | 0.44 |  |
| *LCGN_0239* | Protein of unknown function DUF1211 |  | 0.29 |  |
| *LCGN_0240* | hypothetical protein |  | 0.35 |  |
| *LCGN_0241* | Glyoxalase family protein |  | 0.36 |  |
| *LCGN_0324* | hypothetical protein |  | 0.35 |  |
| *LCGN_0347* | DNA polymerase III epsilon chain |  | 2.05 |  |
| *LCGN_0363* | hypothetical protein |  | 2.41 |  |
| *LCGN_0396* | Predicted esterase | 0.15 | 0.22 |  |
| *LCGN_0397* | Glyoxalase family protein |  | 0.14 | 0.13 |
| *LCGN_0406* | General stress protein Gls24 family |  | 0.34 | 0.43 |
| *LCGN_0427* | hypothetical protein |  | 0.53 |  |
| *LCGN_0482* | Enolase |  |  | 2.10 |
| *LCGN_0484* | LSU ribosomal protein L20p |  | 0.47 |  |
| *LCGN_0491* | hypothetical protein |  | 0.33 |  |
| *LCGN_0503* | hypothetical protein |  | 0.33 |  |
| *LCGN_0506* | PlcB ORFX ORFP ORFB ORFA ldh gene |  | 0.35 | 0.43 |
| *LCGN_0516* | PTS system mannose-specific IIC component |  | 0.17 | 0.15 |
| *LCGN_0589* | hypothetical protein | 4.51 | 3.72 | 6.06 |
| *LCGN_0598* | Lipoprotein signal peptidase |  | 1.96 |  |
| *mtlD* | Mannitol-1-phosphate 5-dehydrogenase |  | 3.64 |  |
| *mtlF* | PTS system mannitol-specific IIA component |  | 3.00 |  |
| *LCGN_0608* | Mannitol operon activator BglG family |  | 3.66 |  |
| *mtlA* | PTS system mannitol-specific IIB component / PTS system mannitol-specific IIC component |  | 3.45 |  |
| *LCGN_0662* | Acetoin dehydrogenase E1 component beta-subunit |  |  | 2.15 |
| *LCGN_0671* | Hydrolase (HAD superfamily) |  | 2.17 |  |
| *LCGN_0677* | phage infection protein |  | 1.96 |  |
| *LCGN_0689* | GTP pyrophosphokinase (p)ppGppsynthetase I |  |  | 2.29 |
| *codY* | GTP-sensing transcriptional pleiotropic repressor codY |  | 0.34 | 0.42 |
| *hepB / ispB* | Heptaprenyl diphosphate synthase component II |  | 0.36 |  |
| *LCGN_0773* | acetyltransferase GNAT family |  | 0.40 |  |
| *LCGN_0813* | hypothetical protein |  | 2.08 |  |
| *fni / idi / yebB* | Isopentenyl-diphosphate delta-isomerase FMN-depeent |  | 2.19 |  |
| *LCGN_0844* | Multimodulartranspeptidase-transglycosylase / Penicillin-biing protein 1A/1B (PBP1) |  | 2.26 |  |
| *LCGN_0845* | SA9H10L |  | 2.46 |  |
| *LCGN_0921* | ABC transporter permease protein |  | 2.36 |  |
| *LCGN_0936* | UDP-N-acetylmuramoylalanyl-D-glutamate--L-lysine ligase |  |  | 2.74 |
| *LCGN_1001* | hypothetical protein |  | 0.40 |  |
| *LCGN_1036* | Transporter |  | 0.44 |  |
| *LCGN_1078* | hypothetical protein |  | 0.39 |  |
| *gpsB* | Cell division protein GpsB coordinates the switch between cylirical a septal cell wall synthesis by re-localization of PBP1 |  | 0.45 |  |
| *LCGN_1115* | hypothetical protein |  | 0.52 |  |
| *LCGN_1181* | hypothetical protein |  | 0.40 |  |
| *LCGN_1229* | hypothetical protein |  | 0.33 | 0.40 |
| *LCGN_1248* | hypothetical protein |  | 2.49 | 3.38 |
| *mscL* | Large-co uctancemechanosensitive channel |  | 0.45 |  |
| *LCGN_1407* | Lipoteichoic acid synthase LtaS Type IIb |  | 2.55 | 3.22 |
| *LCGN_1408* | hypothetical protein |  | 2.68 |  |
| *LCGN_1409* | hypothetical protein |  | 2.38 |  |
| *LCGN_1438* | hypothetical protein |  | 0.37 |  |
| *LCGN_1450* | Aspartate carbamoyltransferase |  | 0.24 | 0.17 |
| *LCGN_1470* | Glucosamine-6-phosphate deaminase |  | 0.51 |  |
| *LCGN_1531* | Nitrilotriacetate monooxygenase component B |  | 0.23 | 0.26 |
| *LCGN_1577* | ImpB/MucB/SamB family protein |  | 1.95 |  |
| *LCGN_1592* | Ribosomal large subunit pseudouridine synthase B |  | 2.36 |  |
| *LCGN_1647* | Nitroreductase family protein |  | 0.24 | 0.26 |
| *ileS* | Isoleucyl-tRNAsynthetase |  | 2.20 |  |
| *LCGN_1707* | hypothetical protein |  | 0.53 |  |
| *LCGN_1747* | hypothetical protein |  | 0.31 | 0.38 |
| *LCGN_1886* | Oxygen-insensitive NAD(P)H nitroreductase / Dihydropteridine reductase |  | 0.29 |  |
| *LCGN_1928* | L-proline glycine betaine bi ing ABC transporter protein ProX / Osmotic adaptation |  | 1.95 | 2.59 |

1. **Other genes differentially expressed exclusively in co-culture.**

| Name | Description | ratio of gene expression in co-culture with high aeration / with low aeration | | |
| --- | --- | --- | --- | --- |
|  |  | DESeq | DESeq2 | EdgeR |
| *LCGN_0029* | hypothetical protein |  | 1.99 |  |
| *LCGN_0030* | S4-domain-containing heat shock protein%3B S4-domain-containing heat shock protein | 2.84 | 2.77 |  |
| *LCGN_0055* | Signal peptidase I |  | 0.52 |  |
| *LCGN_0071* | hypothetical protein |  | 0.32 |  |
| *LCGN_0073* | hypothetical protein |  | 2.03 |  |
| *LCGN_0080* | Glycosyltransferase LafB responsible for the formation of Gal-Glc-DAG |  | 0.43 |  |
| *LCGN_0084* | Preprotein translocase subunit YajC | 2.87 | 2.70 |  |
| *LCGN_0104* | 6-phosphogluconate dehydrogenase decarboxylating |  | 0.40 |  |
| *LCGN_0157* | Glycine betaine ABC transport system ATP-biing protein OpuAA | 0.27 | 0.28 | 0.29 |
| *LCGN_0195* | Flagellar hook-length control protein FliK |  | 0.49 |  |
| *LCGN_0231* | Rrf2 family transcriptional regulator group III |  | 0.60 |  |
| *LCGN_0275* | hypothetical protein |  | 0.51 |  |
| *LCGN_0317* | Cyclopropane-fatty-acyl-phospholipid synthase |  | 2.09 |  |
| *LCGN_0382* | Similar to tetracycline resistance protein |  | 0.23 |  |
| *LCGN_0383* | Phosphopentomutase |  | 0.52 |  |
| *LCGN_0384* | hypothetical protein |  | 0.53 |  |
| *LCGN_0385* | Purine nucleoside phosphorylase |  | 0.58 |  |
| *LCGN_0403* | hypothetical protein |  | 0.49 |  |
| *LCGN_0416* | Cardiolipinsynthetase |  | 0.54 |  |
| *LCGN_0421* | ABC-type multidrug transport system ATPase component |  | 0.27 | 0.29 |
| *LCGN_0422* | ABC transporter permease protein |  | 0.41 |  |
| *LCGN_0423* | Cysteinyl-tRNAsynthetase related protein |  | 0.55 |  |
| *LCGN_0437* | LSU ribosomal protein L27p |  | 0.57 |  |
| *LCGN_0453* | hypothetical protein |  | 0.52 |  |
| *LCGN_0477* | unknown |  | 0.46 |  |
| *LCGN_0501* | hypothetical protein |  | 1.95 |  |
| *LCGN_0580* | Cold shock protein |  | 2.40 | 2.82 |
| *LCGN_0627* | proposed amino acid ligase fou clustered with an amidotransferase |  | 2.02 |  |
| *fabI* | Enoyl-[acyl-carrier-protein] reductase [NADH] |  | 0.55 |  |
| *LCGN_0700* | Inner membrane protein translocase component YidC short form OxaI-like |  | 2.14 |  |
| *LCGN_0703* | Oxidoreductase aldo/keto reductase family |  | 2.03 |  |
| *LCGN_0709* | Aspartyl-tRNA(Asn) amidotransferase subunit C @ Glutamyl-tRNA(Gln) amidotransferase subunit C |  | 2.23 |  |
| *LCGN_0713* | Cold shock protein | 2.75 | 2.61 |  |
| *dut* | Deoxyuridine 5'-triphosphate nucleotidohydrolase | 2.61 | 2.53 |  |
| *LCGN_0734* | hypothetical protein |  | 0.44 |  |
| *LCGN_0833* | ATP-depeent RNA helicase YqfR |  | 1.80 |  |
| *ptcB* | PTS system cellobiose-specific IIB component |  | 1.70 | 1.99 |
| *LCGN_0886* | Inositol-1-monophosphatase |  | 0.48 |  |
| *LCGN_0889* | Cell division trigger factor |  | 2.04 | 2.31 |
| *LCGN_0931* | hypothetical protein |  |  | 2.08 |
| *LCGN_0941* | Neutral e opeptidase O |  |  | 2.31 |
| *LCGN_0968* | fibronectin-biing protein homolog |  | 0.44 |  |
| *dacA / dacB* | D-alanyl-D-alanine carboxypeptidase |  | 0.45 |  |
| *LCGN_0972* | hypothetical protein |  | 0.47 |  |
| *fruR* | Transcriptional repressor of the fructose operon DeoR family |  | 0.20 |  |
| *fruC* | Tagatose-6-phosphate kinase / 1-phosphofructokinase |  | 0.19 |  |
| *fruA* | PTS system fructose-specific IIA component / PTS system fructose-specific IIB component / PTS system fructose-specific IIC component |  | 0.21 |  |
| *fer* | Ferredoxin |  |  | 4.43 |
| *LCGN_1100* | Cold shock protein |  | 2.42 | 2.89 |
| *aroD* | 3-dehydroquinate dehydratase I |  | 0.51 |  |
| *LCGN_1157* | hypothetical protein |  | 0.19 |  |
| *LCGN_1182* | unknown |  | 0.20 |  |
| *LCGN_1183* | hypothetical protein |  | 0.20 |  |
| *LCGN_1237* | NAD(FAD)-utilizing dehydrogenase sll0175 homolog |  | 0.54 |  |
| *LCGN_1247* | hypothetical protein |  | 0.53 |  |
| *LCGN_1262* | hypothetical protein |  | 0.49 |  |
| *LCGN_1276* | Phosphoglycerate mutase |  | 1.62 |  |
| *LCGN_1278* | transcription regulator |  | 1.64 |  |
| *LCGN_1418* | Alpha/beta hydrolase fold |  | 1.94 |  |
| *LCGN_1430* | Isoaspartyl aminopeptidase @ Asp-X dipeptidase |  | 2.07 |  |
| *LCGN_1446* | Gluconate permease Bsu4004 homolog |  | 0.48 |  |
| *LCGN_1452* | Substrate-specific component BioY of biotin ECF transporter | 0.34 | 0.35 |  |
| *LCGN_1475* | hypothetical protein | 0.20 |  | 0.22 |
| *LCGN_1568* | hypothetical protein |  | 2.30 |  |
| *LCGN_1572* | CBS domain protein lmo1865 homolog |  | 2.19 |  |
| *LCGN_1581* | 50S ribosomal subunit maturation GTPaseRbgA (B. subtilis YlqF) |  | 2.22 |  |
| *LCGN_1598* | Dihydrofolate reductase |  | 1.68 |  |
| *LCGN_1600* | ATP-depeent Clp protease ATP-biing subunit ClpX |  | 1.70 |  |
| *engB* | GTP-bi ing protein EngB |  | 1.84 |  |
| *LCGN_1602* | Dihydroneopterin aldolase |  | 2.31 |  |
| *LCGN_1673* | Ribosomal RNA small subunit methyltransferase B |  | 1.91 |  |
| *LCGN_1688* | FtsZ-interacting protein related to cell division |  | 1.81 |  |
| *cysS* | Cysteinyl-tRNAsynthetase |  | 1.99 |  |
| *LCGN_1706* | COG1939: Ribonuclease III family protein |  | 2.20 |  |
| *LCGN_1716* | hypothetical protein |  | 0.54 |  |
| *LCGN_1728* | Hydrolase HAD subfamily IIIA |  | 0.47 |  |
| *argS* | Arginyl-tRNAsynthetase |  | 0.56 |  |
| *LCGN_1784* | UPF0348 protein family |  | 1.62 |  |
| *pheS* | Phenylalanyl-tRNAsynthetase alpha chain |  | 2.33 |  |
| *LCGN_1796* | hypothetical protein |  | 0.46 |  |
| *LCGN_1804* | Zinc protease |  | 0.55 |  |
| *LCGN_1813* | Lactose a galactose permease GPH translocator family |  | 3.39 | 4.15 |
| *LCGN_1865* | Lipid A export ATP-biing/permease protein MsbA |  | 1.63 |  |
