# Supplementary material for: New Insights into the Anti-pathogenic Potential of Lactococcus garvieae against Staphylococcus aureus Based on RNA Sequencing Profiling
Source: Front Microbiol. 2017 Mar 8;8:359. doi: 10.3389/fmicb.2017.00359 (PMC5340753; doi:10.3389/fmicb.2017.00359)
Supplement: Supplementary file 5 [file Table_5.docx]

**Determination of L-threonine impact on *S. aureus* inhibition in microfiltered milk**

Pure culture of *S. aureus* SA15 and co-cultures of *S. aureus* SA15 with *L. garvieae* N201 were performed, under the high or the low aeration level with slight modifications. The cultures were performed in microfiltered milk (Marguerite, Candia, Lyon, France). Catalase at 400 U.mL^-1^ (Sigma) and different concentrations (0 mM, 0.1 mM, 1 mM or 10 mM) of L-threonine (Merck KGaA) were added before the bacteria inoculations. The *S. aureus* growth was evaluated from cultivable cell enumerations after 3 h, 6 h, 9 h and 24 h of incubation by plating as described by Delpech et al. (2015). The whole experimental design was repeated independently three times.

Results are shown in supplementary table 5.
Under the high aeration level, *S. aureus* growth was not significantly inhibited by *L. garvieae* and the L-threonine supplementation had no effect. Under the low aeration, *S. aureus* growth was inhibited by *L. garvieae* at 24 h (difference in *S. aureus* cultivable cell counts of 0.6 log [CFU.mL^-1^] between the pure culture and the co-culture). This low but significant inhibition was not suppressed by the L-threonine supplementation of 100 µM. Moreover, higher L-threonine supplementations (1 mM and 10 mM) did not suppress the inhibitory effect of *L. garvieae* in *S. aureus* growth (data not shown).

## **Supplementary table 5.** Evolution of cell counts **over 24 h in pure cultures or co-cultures of *L. garvieae* N201 and *S. aureus* SA15 in catalase-treated microfiltered milk under a high (shaking) or a low (static) aeration level and supplemented or not with 100 µM of L-threonine. ^a,b,c^ a different letter indicates values which are significantly different (p-value < 0.05 by Newman-Keuls method) through one table row. NT = non-tested values.**

|  | *L. garvieae* presence | - | - | + | + | - | - | + | + |
| --- | --- | --- | --- | --- | --- | --- | --- | --- | --- |
|  | Aeration level | + | + | + | + | - | - | - | - |
|  | Threonine | - | + | - | + | - | + | - | + |
| *L. garvieae* cellular concentration (log [CFU.mL^-1^]) | 0 h | NT | NT | 7.1 ^a^ | 7.1 ^a^ | NT | NT | 7.1 ^a^ | 7.1 ^a^ |
|  | 3 h | NT | NT | 7.3 ^a^ | 7.4 ^a^ | NT | NT | 7.4 ^a^ | 7.4 ^a^ |
|  | 6 h | NT | NT | 7.7 ^a^ | 7.5 ^a^ | NT | NT | 7.8 ^a^ | 7.8 ^a^ |
|  | 9 h | NT | NT | 7.8 ^a^ | 7.7 ^a^ | NT | NT | 7.9 ^a^ | 8.1 ^a^ |
|  | 24 h | NT | NT | 7.9 ^a^ | 7.7 ^a^ | NT | NT | 8.1 ^a^ | 7.9 ^a^ |
| *S. aureus* cellular concentration (log [CFU.mL^-1^]) | 0 h | 6.1 ^a^ | 6.1 ^a^ | 6.2 ^a^ | 6.2 ^a^ | 6.2 ^a^ | 6.2 ^a^ | 6.2 ^a^ | 6.2 ^a^ |
|  | 3 h | 6.6 ^a^ | 6.6 ^a^ | 6.5 ^a^ | 6.6 ^a^ | 6.6 ^a^ | 6.6 ^a^ | 6.6 ^a^ | 6.6 ^a^ |
|  | 6 h | 7.6 ^a^ | 7.6 ^a^ | 7.4 ^a^ | 7.6 ^a^ | 7.6 ^a^ | 7.5 ^a^ | 7.1 ^a^ | 7.2 ^a^ |
|  | 9 h | 8.3 ^a^ | 8.4 ^a^ | 8.0 ^ab^ | 8.0 ^ab^ | 8.0 ^ab^ | 7.9 ^ac^ | 7.2 ^bc^ | 7.2 ^b^ |
|  | 24 h | 8.7 ^a^ | 8.7 ^a^ | 8.7 ^a^ | 8.8 ^a^ | 8.1 ^a^ | 8.2 ^a^ | 7.5 ^b^ | 7.5 ^b^ |
