# Supplementary material for: New Insights into the Anti-pathogenic Potential of Lactococcus garvieae against Staphylococcus aureus Based on RNA Sequencing Profiling
Source: Front Microbiol. 2017 Mar 8;8:359. doi: 10.3389/fmicb.2017.00359 (PMC5340753; doi:10.3389/fmicb.2017.00359)

## Supplementary figure 1. Growth of *S. aureus* in enzymatically treated or untreated supernatants determined by OD600 monitoring. **Growth of *S. aureus* in supernatants prepared from *S. aureus* pure cultures with catalase (solid lines) or from co-cultures with catalase (dotted lines). Supernatants were initially either untreated (A), or treated with proteases (B), or treated with** α**-amylase (C) or treated with lipase (D). Significant differences using the Newmann-Keuls test were indicated by asterisks below the time point (* = p-value < 0.1 and ** = p-value < 0.05).**


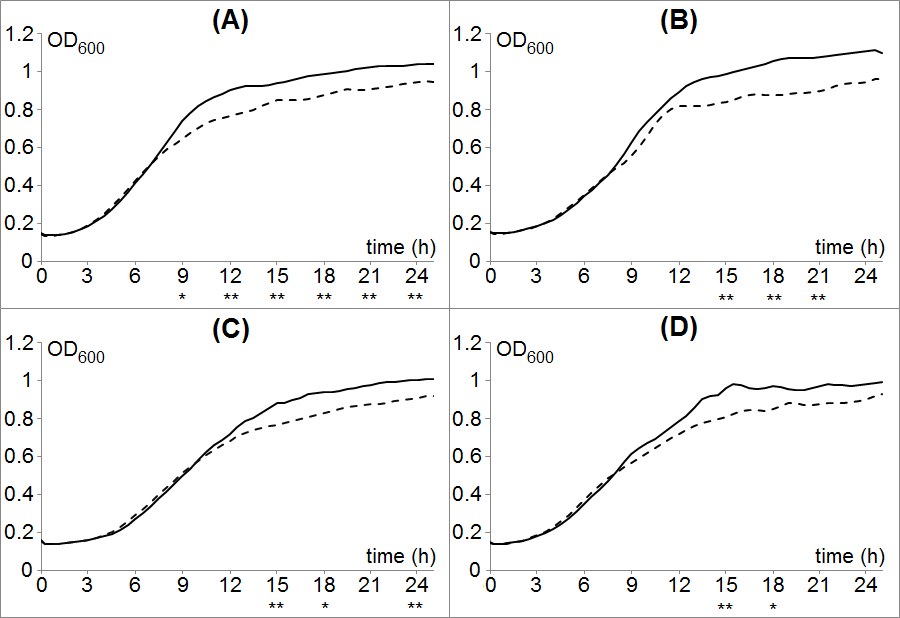

Supplement: Supplementary file 6 [file Data_Sheet_1.docx]
